# Disparities in mobile phone ownership reflect inequities in access to healthcare

**DOI:** 10.1371/journal.pdig.0000270

**Published:** 2023-07-06

**Authors:** Alexandre Blake, Ashley Hazel, John Jakurama, Justy Matundu, Nita Bharti

**Affiliations:** 1 Biology Department, Center for Infectious Disease Dynamics, Penn State University, University Park, Pennsylvania, United States of America; 2 Francis I. Proctor Foundation, University of California, San Francisco, California, United States of America; 3 Kaoko Information Center, Opuwo, Namibia; University of Leeds, UNITED KINGDOM

## Abstract

Human movement and population connectivity inform infectious disease management. Remote data, particularly mobile phone usage data, are frequently used to track mobility in outbreak response efforts without measuring representation in target populations. Using a detailed interview instrument, we measure population representation in phone ownership, mobility, and access to healthcare in a highly mobile population with low access to health care in Namibia, a middle-income country. We find that 1) phone ownership is both low and biased by gender, 2) phone ownership is correlated with differences in mobility and access to healthcare, and 3) reception is spatially unequal and scarce in non-urban areas. We demonstrate that mobile phone data do not represent the populations and locations that most need public health improvements. Finally, we show that relying on these data to inform public health decisions can be harmful with the potential to magnify health inequities rather than reducing them. To reduce health inequities, it is critical to integrate multiple data streams with measured, non-overlapping biases to ensure data representativeness for vulnerable populations.

## Author summary

Mobile phone data are increasingly used to inform public health efforts in both high and low-income settings due to convenience and growing phone penetration. However, digital inequities are ubiquitous and more pronounced in areas where mobile phone ownership is low or heterogeneous. The biases introduced by using mobile phone data to represent populations and their health care needs are rarely measured but have the potential to be detrimental to the most vulnerable segments of populations. We conducted detailed interviews measuring mobile phone ownership, mobility, and access to healthcare in mobile and remote populations in Namibia. We found that mobile phone owners represent a small proportion of the population that is highly mobile and has better access to healthcare. This is likely not unique. Due to the nature of their collection, mobile phone data often underrepresent vulnerable populations. This study demonstrates that uncritically using mobile phone data to inform public health decisions can perpetuate health inequities.

## Introduction

### Background

Human movement and contacts underlie pathogen transmission. Characterizing and quantifying movement greatly improves infectious disease surveillance, control, and prevention efforts. These efforts are particularly important to guide health policies for mobile populations [1–3]. The importance and challenge of measuring movement across spatiotemporal scales has given rise to a broad spectrum of methods to track human movements [4–7]. However, many methods strongly underrepresent or completely miss the most vulnerable populations, which are often most in need of improved public health services. This includes marginalized groups, low-income populations, small or low-density populations, and rural or remote populations.

### Measuring movement

The past decade has seen growing use of novel data sources as proxies for human movement due to technological advances and convenience in the absence of readily available, representative data [8,9]. Current methods to quantify human movement include commercial air traffic [6], satellite derived anthropogenic illumination [5,10], and mobility traces derived from mobile phone call detail records (CDRs) within national boundaries [3,4]. Each of these approaches captures biased samples of populations, though the extent and types of biases vary. Using relatively new data streams to inform public health decisions can leave decision makers with unmeasured biases in population representation, which can harm efforts to improve health equity.

Justified by a broad temporal increase in the usership of mobile phones, CDRs in particular are increasingly used as proxies for human movement [9,11–13]. CDRs are collected for billing purposes from all mobile phones regardless of device capabilities, unlike GPS data or app-derived information. CDRs reflect mobile phone usage and document the towers that route each telecommunication transaction (call, text, or other billable event). Despite usership growth, mobile phone penetration is substantially lower in low-income countries when compared to high-income countries [14] and phone usage is heterogeneous in many low-income and under-resourced populations within middle- and high-income nations [15,16]. In 2022, phone ownership for individuals older than ten years was 92.9% in Europe and 88.5% in the Americas, and 60.6% in Africa [17]. Relentless advances in mobile communications technology have, in many ways, widened this gap and increased inequities that stem from access to technology. This is because technological advances have not improved access to phones but they have increased the advantages of phone ownership; this digital divide is associated with rurality, lower literacy levels, and lower gross domestic product at the national level [18], with additional subnational disparities [19,20].

**Fig 1:**
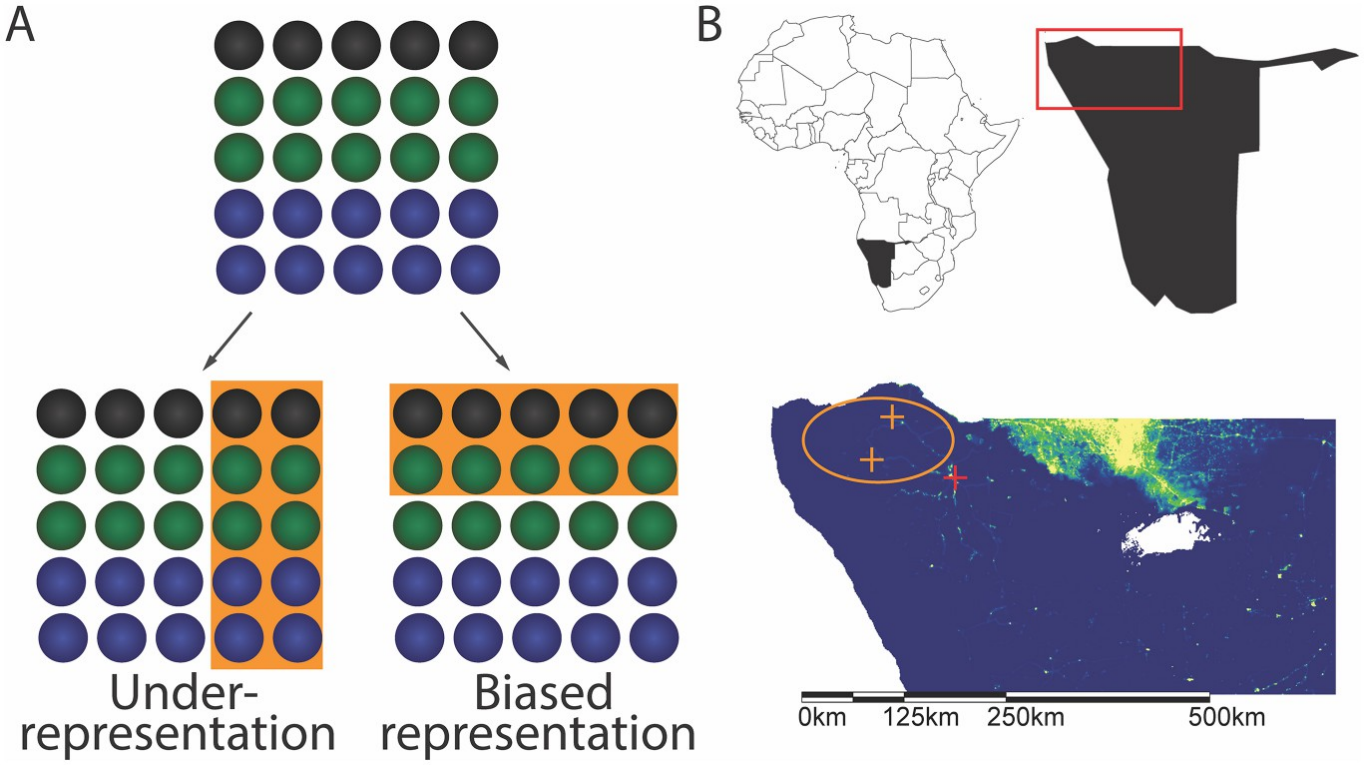
Overview of concept and location. (**A**) Schematic showing underrepresentation and biased representation of a population. Circles represent individuals; black, blue, and green colors each represent one of three possible values for a demographic characteristic. Orange shaded areas represent individuals selected for inclusion in each data set. (**B**) Above: Inset map of continent, Namibia in black, and region of interest outlined in red. Below: Detailed map of the region of interest showing population density in 2016 (yellow = high; blue = low; white = NA (Etosha National Park, no human population)). Study area circled in orange, health clinics (orange crosses), regional hospital (red cross). Map created in ArcGIS with data from GADM (https://gadm.org/).

Phone data analyses often incorrectly assume a 1:1 relationship between a person and a phone [21]. Some studies explore scalable solutions to correct for low ownership but cannot address biased usership [22]. The necessary anonymity of mobile device data renders it impossible to assess potential biases from the data itself (Fig 1A). This can result in unknowingly ignoring complex ownership and usership patterns. Additionally, the mobility patterns of mobile phone users cannot be tracked in areas that lack mobile phone reception, which is frequently the case in low-income, low population density areas. Overall, this leads to misrepresenting mobility patterns and underrepresenting the segments of populations most in need of improved health services. Public health efforts that are intended to reach the most vulnerable and least resourced members of populations that rely on biased data can result in interventions that are counterproductive to improving health equity. These populations are often small in size, which contributes to the reasons they are routinely overlooked. Regardless of their size, the most vulnerable populations define global health equity, and they must be prioritized instead of forgotten.

### Phones and health in Namibia

Namibia is a middle-income nation with a relatively high infectious disease burden. The top ten causes of death include HIV/AIDS, lower respiratory infections, tuberculosis, and diarrheal diseases [23]. Public health efforts in Namibia increasingly incorporate phone-derived data, specifically CDRs, to measure movement to understand spatial connectivity, trip duration, and seasonality to inform infectious disease prevention and management, including malaria elimination and HIV risk reduction [24–27]. In 2013, mobile phone ownership in Namibia was estimated at 95% in urban areas [28], with reliable network coverage. However, populations in Namibia vary greatly in size and density (Fig 1B). In Namibia’s non-urban areas, where health improvements are needed most, phone ownership and network coverage had not been measured prior to this study. Additionally, mobile and remote populations are not consistently included in traditional censuses in the region [29,30].

The Kunene province is a desert area in northwestern region of Namibia (Fig 1B). Local residents are largely nomadic pastoralists who move seasonally and many are members of the Himba tribe. Residents travel primarily by walking. The area has a minimal, informal road network, and scarce access to petrol. There is a strong gender division of labor in these populations: men herd cattle and women manage childcare and subsistence farming. As a result, men travel greater distances and move more frequently than women do and men maintain control over valuable assets, specifically livestock. These nomadic populations represent vulnerable groups for both preventable and emerging infectious diseases. Although basic public healthcare is relatively affordable and accessible in urban areas of Namibia, in remote areas, facilities are scarce and distance to the nearest clinic is often prohibitive. In populations with limited access to healthcare, many illnesses go undiagnosed, untreated, and unreported [31].

To assess data representativeness in mobile phone-derived data in non-urban areas, we conducted detailed interviews among residents of the Kunene province (Fig 1B) in 2015 (rainy season) and 2016 (dry season). We collected data to compare characteristics of mobile phone owners and non-mobile phone owners among participants. Specifically, we assessed demographic characteristics (gender and age), self-reported recent movements and travel, and measures of access and barriers to healthcare (recency, frequency, distance, monetary cost, time cost) (see Supporting Information in S1 Text for survey details and Table O in S1 Text for survey instrument). We find that data derived from mobile phones in this remote population: 1) underrepresent women, 2) overestimate mobility and access to care, and 3) underrepresent remote and rural areas. Using mobile phone-based data to inform public health needs would largely overlook the segments of the population that need improvements the most. Instead, measuring biases in phone usage and integrating multiple data sources with non-overlapping biases can help include the most vulnerable members of populations in data that are used to inform public health efforts. This is an important step towards improving health equity, a priority for The United Nations’ (UN) Sustainable Development Goals (SDG) [32].

## Material and methods

### Interviews

#### Sampling methods and recruitment

We conducted interviews in two settlements in the desert of Kaoko in the Kunene region, Namibia, in February of 2015 (rainy season) and October of 2016 (dry season) (Fig 1B). The vast majority of participants self-identified as members of the Himba tribe. Participants also included members of the Tjimba, Ovambo, Zemba, and Twe tribes. The Himba are the majority tribe in Kaoko, and all tribes in the area are Bantu-speaking and participate in the cattle-herding culture and economy. The lifestyle is highly mobile and semi-nomadic, with pastoralism supported by subsistence agriculture. We conducted interviews at the same two physical settlements across two years, and participants included residents of these settlements as well as visitors. These data represent mobile individuals and families from many locations and settlements within the Kaoko region of northern Namibia.

Interviews were performed in the local language, Otjiherero, with a translator who was the same gender as the participant.

#### Inclusion criteria

Study participants were restricted to adults. The designation of “adult” was locally determined by household responsibilities, interpersonal relationships (sexually active), or age, in instances when it was known. Locally, individuals are considered adults at approximately 16 years of age.

Pregnant women could participate in the study, as it posed no risk to the mother or fetus.

Participants were required to provide consent to participate in the study and could revoke their consent at any point.

#### Sample size

We conducted a total of 167 interviews. We conducted interviews with 102 adults in 2015. Twenty-five of these (12 men, 13 women) were completed in a short format during a pilot phase during which we primarily collected demographic data. We conducted full-length interviews with 75 adults (37 men, 38 women). The remaining 2 adults (2 women) provided basic demographic information as child guardians. In 2016, we conducted full-length interviews with 65 adults (31 men, 34 women). Eight adults were interviewed in both 2015 and 2016.

#### Instrument items analyzed in this study

This study analyzes some of the data collected during the interviews. Included here are participants’ answers to questions about phone ownership, phone use during their lifetime, areas with phone reception, travel time to the nearest health center (discrete values in hours; estimated by participants in hours or based on sun positions), travel destinations in the previous 12 months (up to 5), mode of travel to the nearest health care center, and the ability to access health care when wanted. Participants were also asked about basic demographic information, individual and household resources, and sexual contacts in the previous 12 months (Table O in S1 Text contains the full survey instrument).

### Statistical analysis

All analyses were done using R 4.0.3 [33]. We provide a basic description of the participants with means and percentages before multiple imputations (Table 1 and Table A in S1 Text). We addressed data missingness with multiple imputations (15 imputations) and analyzed every imputed data set before applying the Rubin rules to pool the estimates of interest [34,35] (see S1 Text). All the tests performed to compare groups (men vs women, participants interviewed in 2015 vs participants interviewed in 2016, or mobile phone owners vs non-owners) were performed using the imputed data sets. We calculated differences in means and in proportions in every imputed data set, pooled them together, and obtained p-values using Wald tests [35].

**Table 1:** Summary of the characteristics of the participants including mobile phone ownership. Overview of participant characteristics before applying multiple imputations.

	Participants (N = 159)		Mobile phone owners (N = 41)		Non-phone owners (N = 91)		NA for phone ownership (N = 27)	
	n	%	n	%	n	%	n	%
**Year**								
**2015**	102	64.2	26	63.4	49	53.8	27	100
**2016**	57	35.8	15	36.6	42	46.2	0	0
**Gender**								
**Women**	85	53.5	11	26.8	59	64.8	15	55.6
**Men**	74	46.5	30	73.2	32	35.2	12	44.4
**Age**								
**16–25**	36	22.6	10	24.4	23	25.3	3	11.1
**26–35**	41	25.8	18	43.9	22	24.2	1	3.7
**36–45**	31	19.5	6	14.6	18	19.8	7	25.9
**46–59**	24	15.1	5	12.2	12	13.2	7	25.9
**60+**	27	17.0	2	4.9	16	17.6	9	33.3

We used a principal component analysis to identify a reasonable proxy for access to health care. We applied it to a set of variables that addressed access to care and checked their loadings [36]. The set of variables included the number of travel destinations, travel to health care by car or another mode of transportation, the travel time to access healthcare, and the ability to access health care when desired. We used the loadings to identify the variable most strongly correlated with the main principal component and used that variable as a proxy for access to care in the rest of the analysis (see S1 Text).

We compared access to care between mobile phone owners and non-phone owners by calculating the mean values of the identified proxy for each group and pooling the differences in means of every imputed data set. To minimize confounding biases in this comparison, we calculated propensity score and calculated the difference in means after trimming and matching in every imputed data set (Figure B in S1 Text) before pooling the estimates to ensure that mobile phone owners and non-owners were as similar as possible [37] (see S1 Text, and Figure B in S1 Text).

We investigated the bias in access to care by estimating the distribution of the identified proxy among phone users only and for the whole sample by calculating the difference between the two groups. We calculated the probability mass for every value after applying Rubin rules to the imputed data sets by gender and mobile phone ownership and smoothed it with discrete kernel density estimators to minimize random noise [38,39]. We then estimated the distribution of the proxy among mobile phone owners as well as for the total population by calculating the weighted average of the smoothed distribution by gender and mobile phone ownership with pooled proportions from imputed data sets used as weights. Little to no difference between the two probability masses would point toward no bias. Conversely, a positive or a negative difference (with the distribution of mobile phone owners as a reference) point toward an over- or underrepresentation in mobile phone-based data. We calculated the 95% CI of the average value of the proxy in mobile phone owners and for the total population after making single imputations embedded in bootstrap (1000 samples) with the percentile method [40].

We assessed the bias in movements that would be captured by mobile phone-based data by participant mobile phone ownership and by analyzing the distribution of the numbers of recently visited locations, including travel destinations (up to 6 reported per participant) and home locations. We classified all destinations according to mobile phone network reception availability based on our observations in the field with MTC mobile phones, the main service provider in Namibia, and participants’ responses to questions about locations with and without phone reception. We classified destinations as follows: A) areas where phone reception was easily and widely available, B) areas with limited access to phone reception, including where reception was only available at elevation and required significant walking in mountainous terrain to reach, and therefore not accessible to everyone, C) areas with no access to mobile phone network reception, or D) areas with unlikely access to phone reception but for which we lacked definitive information on the presence or absence of reception. We calculated the percentage of participants’ destinations and visits that would not be captured by mobile phone-based data.

### Ethics

The study design has approval from Penn State’s Institutional Review Board (IRB #STUDY00001510: Movement and Pathogens in Namibia) and Institutional Biosafety Committee (IBC #48898). Each interview began with an explanation of the survey process and purpose of the research. Data collection began after participants provided their formal verbal consent. Participants could decline to answer or skip any questions, decline continuation at any point during the survey, or revoke consent. Prior to conducting field work, the authors obtained research visas from the Namibian Ministry of Health and Social Services (MOHSS) and local institutional support through The University Center for Studies in Namibia (TUCSIN).

## Results

### Overview

Participant characteristics from interviews are presented in Table 1 and Table A in S1 Text. About two thirds of the total participants were interviewed in 2015 (102/167) and the remaining third were interviewed in 2016 (65/167). The gender ratio of participants was slightly in favor of women (80 men and 87 women, or 52.1% of women). About half of all participants were between 16 and 35 years old (49.1%), though participant age distribution varied by year; 23.5% vs 6.2% were aged 60+ years during 2015 and 2016, respectively. Out of all the participants who answered questions about mobile phone ownership, only 31.4% (44/140) of the participants reported owning a phone and 58.6% (82/140) reported that they had used a phone in their lifetime. 73.2% (101/138) of participants answered that they were unable to access a health care center when they wanted medical attention.

For the remaining results, we calculated three estimates due to the lack of independence of the data collected from the eight individuals who were interviewed in both 2015 and 2016: A) considering their responses in 2015 and 2016 as independent data points, B) excluding their data collected in 2015, and C) excluding their data collected in 2016. These estimates were very similar. When analyzing data from both collection years for the population, we include only the data collected in 2015 for these eight individuals. The resulting sample size is 159 unique participants, and 41 unique mobile phone owners (Table 1) (see S1 Text for full details and other estimates).

Mobile phone ownership was more frequent among participants interviewed in 2015 compared to 2016, 34.7% (26/75) vs 27.7% (18/65) (Table 1). The difference was not statistically different when tested on imputed datasets (p = 0.54). However, the proportion of participants who reported having ever used a mobile phone in their lifetime was lower in 2015 than in 2016; 48.0% (36/75) vs 70.8% (46/65) (Table A in S1 Text). This difference was statistically significant when tested on imputed datasets (p<0.01). Overall, phone ownership and usership were significantly greater in men compared to women; 48.4% (30/62) of men vs 15.7% (11/70) of women reported phone ownership, and 75.8% (47/62) of men vs 42.9% (30/70) of women reported prior phone usership in their lifetime (Table 1 and Tables A and B in S1 Text). These differences were statistically significant when tested on imputed datasets (respectively p<0.001 for phone ownership and p<0.001 for phone usership).

**Fig 2:**
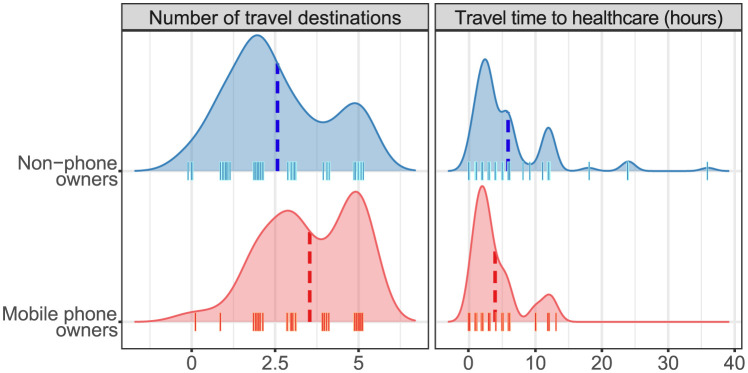
Distributions of the number of travel destinations and travel time to healthcare for non-phone owners (blue) and mobile phone owners (red). The curves show density plots of the distributions of the number of travel destinations and travel time to healthcare (no data were imputed for these density plots), with a jittered rug below. The mean values are indicated by dashed vertical lines.

Phone owners traveled to a greater number of destinations than did non-phone owners in the 12 months preceding the interview, reporting means of 3.5 destinations vs 2.6 destinations (Fig 2). When pooling estimates on imputed datasets the mean number of destinations were.3.1 vs 2.1 respectively (p<0.01). When stratified by gender, this difference in travel history among phone owners was statistically significant in women (p<0.05) but not in men. Phone owners also experienced significantly shorter travel times to health care centers compared to non-phone owners, reporting means of 3.8 hours vs 5.7 hours in transit, respectively (p<0.05). When stratified by gender, the association between phone ownership and shorter travel times to health care centers remained significant in men (p<0.05) but not in women, among whom phone ownership was very low (Figures C and D and Tables C and D in S1 Text).

To reduce the dimensionality of the data, we applied a principal component analysis (PCA) to all the previously specified variables that were related to access to health care. The travel time to a health care center was identified and then used as the main proxy for access to care for the remainder of the analysis (see S1 Text, Figure A in S1 Text).

### Biased estimate of access to care

**Fig 3:**
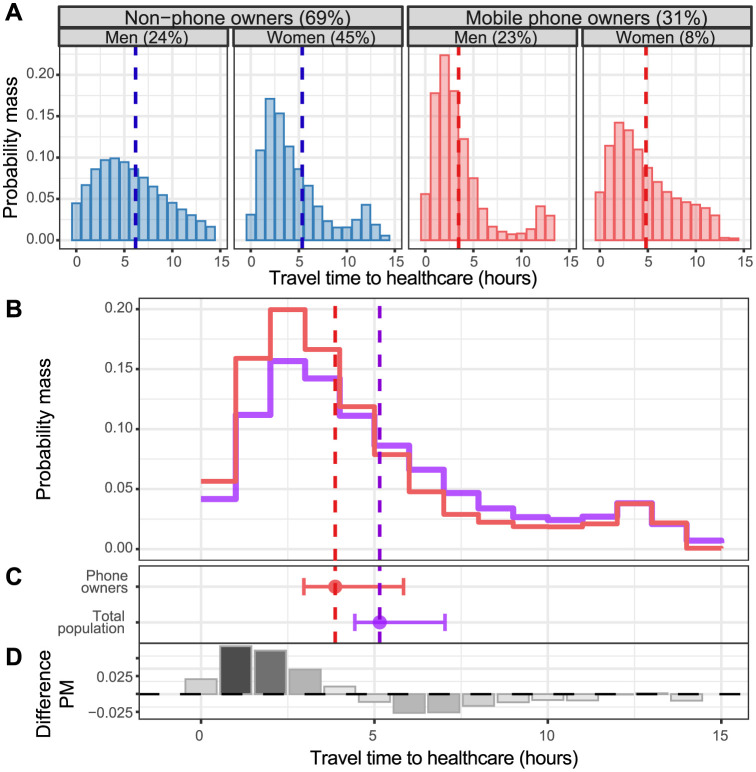
Distributions of travel time to healthcare by gender and mobile phone ownership. (**A**) Distribution of travel time to healthcare by gender and mobile phone ownership (non-phone owners in blue and mobile phone owners in red) after applying a discrete kernel density estimator [38,39] on the distribution after multiple imputation (truncated for values above 15 hours). The dashed vertical lines show the average travel time to health care for each group after multiple imputations. (**B**) Distribution of travel time to healthcare considering only mobile phone owners (red) and total population (purple). Distributions are the results of a weighted average of the distribution in B using proportions of each category shown in A after multiple imputations. The dashed vertical lines show the average travel time to healthcare for mobile phone owners (red) and for the total population (purple) after multiple imputations. (**C**) Average travel time to healthcare for mobile phone owners (red filled circle) and for the total population (purple filled circle) after multiple imputations and their 95% CI estimated by bootstrap. (**D**) Difference in the distribution of travel time to healthcare between mobile phone owners and the total population (the reference group). This is the difference of the two step lines displayed in B. For travel times to healthcare that reported more frequently by mobile phone owners than by the total population, the values are shown as positive. Light shades of grey represent smaller absolute differences; dark shades of grey represent greater absolute differences.

The smoothed distribution of travel time to healthcare highlighted a greater probability of shorter travel times among mobile phone owners, which was even more pronounced among men (Fig 3A). The average values for travel time to healthcare among mobile phone owners compared to the total population was 3.9 hours (95% CI: 3.0–5.8) vs 5.2 hours (95% CI: 4.4–7.0), respectively (Fig 3C). The smoothed distribution of travel time to healthcare taken from mobile phone owners would over-represent values below 5 hours and mostly under-represent values over 5 hours (Fig 3B and 3D).

The reduction in mean travel time after propensity score matching was consistent with greater access to care among phone owners and was close to significance, -2.6 hours of travel time to healthcare (95%CI: -5.5–0.3) (Tables I and J and Figure E in S1 Text).

### Biased representation of mobility

**Fig 4:**
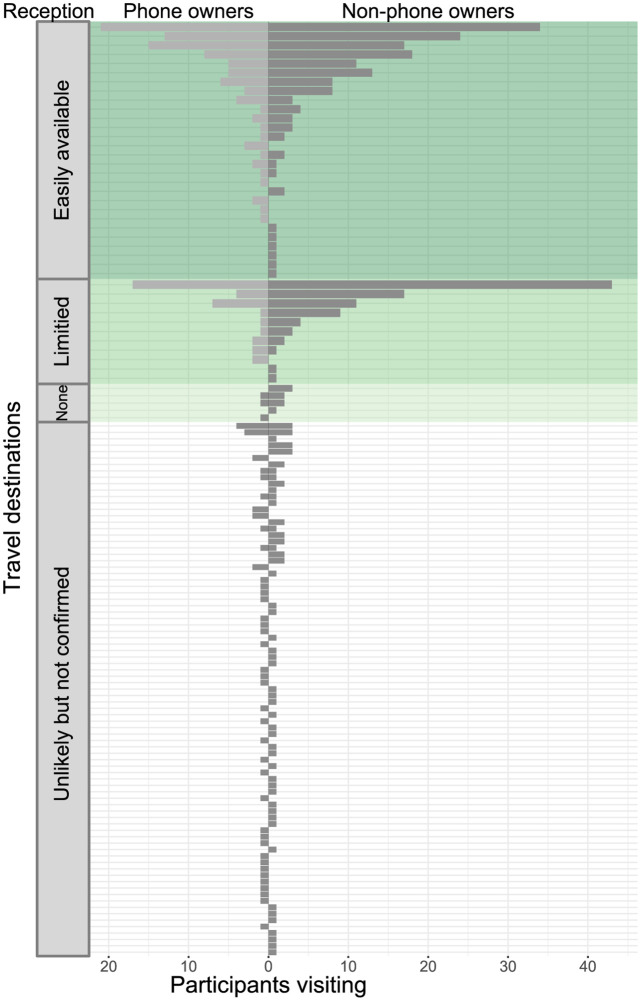
Distribution of participant destinations by mobile phone network reception and mobile phone ownership. Each row represents a unique town and the length of each bar represents the number of participants who reported traveling there (no data were imputed for this histogram). Locations are divided vertically into categories based on mobile phone network reception. Visitor numbers are divided horizontally by visitors’ mobile phone ownership (186 destinations from 41 mobile phone owners on the left, 325 destinations from 91 non-phone owners on the right, 25 destinations from the 27 participants with missing values for mobile phone ownership are not shown). Visits that would not be captured by mobile phone-based data are shown in dark grey, visits that would be captured by mobile phone-based data are shown in light grey.

Mobile phone derived data can only capture movements of mobile phone owners in areas with mobile phone network reception. The 41 participants who owned mobile phones (25.8% of 159 participants) reported 34.7% (186/536) of all 536 reported recent travel destinations (including their home settlements) (Fig 4).

Access to mobile phone reception did not differ significantly between the destinations of mobile phone owners and non-phone owners (Fig 4, Table L in S1 Text); 50.3% (257/511) of all visits were to areas with widely available network reception, 25.2% (129/511) of visits were to areas with limited access to network reception, 2.2% (11/511) of visits were to areas with no access to network reception, and 22.3% (114/511) of visits were to areas with unlikely but unconfirmed access to network reception. Although the access to network reception was similar between the destinations reported by mobile phone owners and non-phone owners, 73.8% (377/511) of the reported destinations would be missed by mobile phone-based data (Fig 4). Of the missed visits, 33.2% (125/377) were to areas with no access or unlikely but not confirmed access to mobile phone reception. Visits to areas with no phone reception, or unlikely but unconfirmed reception represented 24.5% (125/511) of all travel destinations reported by participants who answered questions related to mobile phone ownership.

## Discussion

This study presents original, detailed data on a small number of people but comprehensively represents the populations interviewed. These data represent understudied populations that are often missed in routine data collection on demographics and health, have limited access to health care, and are not prioritized by current health policies. We minimized the effect of the small sample size by applying appropriate statistical approaches to avoid dropping data points and to minimize confounding factors.

With mobile phones being increasingly used in public health data collection [8,41,42], we measured biases in mobile phone-derived data that are often acknowledged, but rarely quantified, and frequently ignored. Our analysis presents evidence that phone owners have better access to health care and greater mobility than members of the same population who do not own phones. We also show that mobile phone-based data provide a skewed perception of local mobility patterns. These data miss a majority of reported travel destinations due to low phone ownership. They also fail to include movement to areas without phone network reception, which tend to overlap with areas that are most in need of improved access to public health resources. Further, using mobile phone data to track the spread of a transmissible pathogen in a region like this one would miss most movements and contacts. This strategy would be highly misleading and ineffective for outbreak response efforts hoping to control the number of new cases and limit the spatial spread of a transmissible pathogen [21].

Mobile phone ownership in remote populations of Himba pastoralists in Namibia was significantly more prevalent in men than women. Mobile phone owners reported much shorter travel times to health care centers compared to non-phone owners, as well as more time-efficient travel methods. Representing these populations with data collected from mobile phone-owners overestimates travel destinations per person and underestimates travel time to a health care center per person. Using mobile phone data to represent this population prevents the detection of the very inequities that require improvement. Failing to measure data biases while relying on biased data to guide policies reinforces existing health inequities.

Many low and middle-income countries (LMIC) report rapid growth in mobile phone usage and infrastructure [18,20]. It is often expected that this trend will continue globally and that low or biased phone usership will simply be overcome with time. Our data suggest that universal growth of mobile phone ownership and infrastructure is unlikely; these interviews were conducted 19 months apart and phone ownership rates were constant and low, with a slight decrease over time. Mobile phone network coverage in the area also did not improve during this time. Subsequent visits to this area and these populations indicate that phone reception and ownership have not increased in recent years.

Although these populations are small in size and number, addressing health inequities for members of the Himba tribe and other underrepresented populations is necessary for public health progress. Remote populations often make up only a small percentage of national or regional populations, and working with these populations necessarily yields small sample sizes. However, ignoring small populations in public health efforts propagates cycles of underrepresentation in data. Remote populations play an important and often underappreciated role in the transmission, emergence, and persistence of infectious diseases [43,44]. Overlooking these groups hinders the final stages of elimination of vaccine preventable transmissible pathogens like polio and measles. In addition, a lack of access to basic health care in remote areas leads to delayed outbreak detection of endemic infections and emerging pathogens [45], which can increase morbidity and mortality. The UN emphasizes the global importance of equitable access to health care as a basic human right for all. Several of the SDGs prioritize improvements in health equity as critical milestones towards progress [32,46].

Large data sets cannot overcome inherent biases by virtue of their size alone [47]. Gender, wealth, education, and disability create gaps in phone ownership at various levels of aggregation across low and middle income countries (LMIC) [15,16,48,49]. These characteristics are also associated with differential access to healthcare [50,51]. Biases in population representativeness in CDRs are often estimated by comparison to another source in which data are aggregated spatially or reported at the population-level instead of the individual-level, such as income subgroups [52–54]. Unfortunately, it is very difficult to identify biases in data representativeness in aggregated data, because heterogeneities and inequities often intersect and are present at fine scales. Traditional data sources, such as population-based surveys, also contain biases in remote areas. When data collection methods have been in use for a long time, their biases become relatively well understood and, once detected, data collection efforts can be adjusted to reduce or account for biases in data representativeness. For example, this study collected data at the individual level. This level of granularity allowed us to measure biases while avoiding the uncertainty associated with comparison data that could be lost by aggregating across administrative areas, age groups, or income levels [52,53].

The Kunene region is not unique regarding remoteness, heterogeneous mobile phone ownership, and wireless signal coverage scarcity. Interviews that are specifically designed to assess populations in remote areas are labor, time, and cost intensive. However, they are critical to identify and monitor populations in need of public health improvements and to assess their representation in proxy measures, including mobile phone data. Surveys that collect data on both phone ownership and variables of interest in targeted areas make it possible to measure biases at comparable, operational scales. High granularity data collected from a source unrelated to mobile phones, such as surveys, are necessary to correct biased estimates of mobility from phones. Alternatively, some studies have successfully used GPS devices paired with surveys, predicated on an understanding of the acceptance and usefulness of GPS devices in a local context [55–58]. This approach is advantageous because it is not impacted by phone network reception or differential mobile phone ownership. Unfortunately, unpredictable long-distance travel over long time periods made GPS device retrieval impossible and we were unable to execute this strategy in northern Namibian pastoralist populations. Integrating multiple data sources improves estimates of the sizes and movements of mobile populations [59], improves data quality on health indicators, and can help guide decision makers towards reducing health inequalities. This strategy also augments the interpretability and usability of data streams like CDRs while minimizing their potential drawbacks.

Well-informed health policy decisions produce effective and equitable improvements in population health outcomes [60,61]. Prioritization is critical in resource-limited settings, as is impact assessment [62,63]. Barriers to implementing effective health policy include inaccurate and biased assessments of the underlying problems and the impacted populations [64]. Accurate estimates of the health needs of mobile or remote populations are critical for reducing the burden of infectious diseases and improving global health. Addressing problems of data representativeness is critical to ensure data sources do not become tools that enhance inequities.

Health care that serves all populations, no matter how small or remote, is integral to the management of infectious diseases. Pathogens persist in areas with insufficient disease surveillance and prevention [65]. Decisions based on data that have not been critically assessed for representativeness and biases can promote dangerous health inequities by incorrectly assuming inclusion of the least visible groups.

## Supporting information

S1 Text Supporting information providing additional details on the methods and results. (DOCX)
