# Supplementary material for: Disparities in mobile phone ownership reflect inequities in access to healthcare
Source: PLOS Digit Health. 2023 Jul 6;2(7):e0000270. doi: 10.1371/journal.pdig.0000270 (PMC10325035; doi:10.1371/journal.pdig.0000270)
Supplement: S1 Text — (DOCX) [file pdig.0000270.s001.docx]

Supporting Information for

**Disparities in mobile phone ownership reflect inequities in access to healthcare: S1 Text**

Alexandre Blake *et al.*

Corresponding author Nita Bharti. Email: nita@psu.edu

**Methods**

**Additional information on data collection**

**Instrument and data collection**

Each interview began with an explanation of the survey process and purpose of the research. All explanations and questions were translated from English to Otjiherero, evaluated for clarity and cultural context, and back-translated from Otjiherero to English by two team members who were native Otjiherero speakers, and who also translated during the interviews. Adjustments were made as necessary to ensure consistent interpretations. Participants were encouraged to ask questions for clarification, decline to answer or skip any questions, or decline continuation at any point during the survey.

**Herpes Simplex Virus detection**

As a measure of transmissible pathogens, we also collected biological samples to test for Herpes Simplex Virus (HSV) shedding. While prevalence varies in this region [1], HSV is a commonly found and directly transmitted virus via both oral (HSV1) and sexual (HSV2) transmission routes and causes lifelong infection. Oral HSV infection was used as a proxy for pathogens that can be transmitted by casual contact.

Oral and/or genital swabs were self-collected by survey participants with instruction from researchers and placed on Whatman FTA cards. Extraction and sequencing of HSVs were performed at Penn State University (manuscript in preparation). The detection of HSV DNA from a participant indicated that they were shedding virus and infectious at the time of sample collection.

**Additional information on the statistical analyses**

**Multiple imputations**

We performed multiple imputations by chained equations [2,3] assuming data were missing at random. We selected the variables related to individual characteristics we used in the analysis and generated 15 imputations before applying Rubin’s rule to pool the estimates from every imputed dataset.

We obtained p-values when comparing means or percentages using Wald tests to compare the test statistic assuming the null hypothesis and an F distribution with one and v degrees of freedom, with v calculated based on the relative increase of variance due to data missingness [4,5].

**Principal component analysis of the variables related to mobility and access to care**

We used principal component analysis (PCA) to identify the most relevant variable to use as a proxy for access to health care. We included the four variables that reflected the ability to travel to and access a health care center (number of travel destinations, ability to travel to a health care center using a car, travel time to access a health care center, and ability to access a health care center) and applied PCA on the whole dataset as well as on each data collection year stratum (Figure A, panel A). The first and the second principal components (PC1 and PC2, respectively) reflected the ability to access health care and mobility, based on the loadings of each variable (Figure A, panel B). PC1 explained 92.7%, 90.2%, and 91.3% for 2015, 2016, and both years combined (Figure A). The loading of the travel time to a health care center was systematically close to 1 or -1 for PC1, respectively 0.997, -0.999, and 0.999, whereas the other variables had a loading close to 0. We then used the travel time to a health care center as the main proxy for access to health care.

Figure A: Principal component analysis applied to variables related to mobility and access to health care. (A) Bar plot displaying the percentage by variance explained by each principal component. (B) Scatter plot displaying the loadings of each variable for the first two prinicipal components with the color indicating the sign (blue circles = negative loading, grey circles = loadings cloe to 0, and red circles = positive loadings).

**Trimming procedure**

Trimming consisted of fitting a logistic regression model with phone ownership as the outcome, predicting the probability of phone ownership, and removing individuals until the range of the phone owning probability between phone owners and non-phone owners had common support. The trimming was repeated until no individual needed to be removed after iteratively refitting the model and recalculating phone ownership probability.

For every imputation, the trimming procedure followed an iterative process repeating the following steps:

1. Fit a logistic regression model with mobile phone ownership as dependent variable
2. Estimate the probability of owning a mobile phone, the propensity score
3. Remove
   1. The participants who own a mobile phone with a propensity score above the highest propensity score of the participants who do not own a mobile phone
   2. The participants who do not own a mobile phone with a propensity score below the lowest propensity score of the participants who own a mobile phone

Those steps are repeated until no more participants are excluded.

The scaled density of the propensity score of mobile phone owners and non-phone owners for all 15 imputations at the end of the trimming procedure is presented in Figure B.

Figure B: Density plot of the predicted probability of owning a phone after the trimming procedure in 15 imputations.

**Results**

**Description of the participants**

The characteristics described in the manuscript are a subset of a larger data collection effort during the interviews. Table A describes the characteristics of the participants and includes additional variables, such as HSV shedding, travel method to a health care center, and reporting at least one deceased child.

Table A: Description of the characteristics of the participants with data collection year and gender. Percentages and means are calculated on raw data (without multiple imputations).

|  | **Recruited in 2015 and 2016^ab^**  **(N=159)** | | |  | **Recruited in**  **2015**  **(N=102)** | |  | **Recruited in**  **2016**  **(N=65)** | |  | **Women^a^**  **(N=85)** | |  | **Men^a^**  **(N=74)** | |
| --- | --- | --- | --- | --- | --- | --- | --- | --- | --- | --- | --- | --- | --- | --- | --- |
|  | **n** | | **Percentage or mean** |  | **n** | **Percentage or mean** |  | **n** | **Percentage or mean** |  | **n** | **Percentage or mean** |  | **n** | **Percentage or mean** |
| **Recruitment year** |  |  | |  |  |  |  |  |  |  |  |  |  |  |  |
| **2015 (Rainy season)** | 102 (102) | 64.2 (61.1) | |  |  |  |  |  |  |  | 53 | 62.4 |  | 49 | 66.2 |
| **2016 (Dry season)** | 57 (65) | 35.8 (38.9) | |  |  |  |  |  |  |  | 32 | 37.6 |  | 25 | 33.8 |
|  |  |  | |  |  |  |  |  |  |  |  |  |  |  |  |
| **Age group** |  |  | |  |  |  |  |  |  |  |  |  |  |  |  |
| **16-25** | 35 (38) | 22.0 (22.8) | |  | 19 | 18.6 |  | 19 | 29.2 |  | 22 | 25.9 |  | 13 | 17.6 |
| **26-35** | 41 (44) | 25.8 (26.3) | |  | 19 | 18.6 |  | 25 | 38.5 |  | 16 | 18.8 |  | 25 | 33.8 |
| **36-45** | 31 (32) | 19.5 (19.2) | |  | 20 | 19.6 |  | 12 | 18.5 |  | 20 | 23.5 |  | 11 | 14.9 |
| **46-59** | 24 (24) | 15.1 (14.4) | |  | 20 | 19.6 |  | 4 | 6.1 |  | 15 | 17.6 |  | 9 | 12.2 |
| **60+** | 27 (28) | 17 (16.8) | |  | 24 | 23.5 |  | 4 | 6.2 |  | 12 | 14.1 |  | 15 | 20.3 |
| **Missing value** | 1 (1) | 0.6 (0.6) | |  | 0 | 0 |  | 1 | 1.5 |  | 0 | 0 |  | 1 | 1.4 |
|  |  |  | |  |  |  |  |  |  |  |  |  |  |  |  |
| **Gender** |  |  | |  |  |  |  |  |  |  |  |  |  |  |  |
| **Women** | 85 (87) | 53.5 (52.1) | |  | 53 | 52.0 |  | 34 | 52.3 |  |  |  |  |  |  |
| **Men** | 74 (80) | 46.5 (47.9) | |  | 49 | 48.0 |  | 31 | 47.7 |  |  |  |  |  |  |
|  |  |  | |  |  |  |  |  |  |  |  |  |  |  |  |
| **Mobile phone ownership** |  |  | |  |  |  |  |  |  |  |  |  |  |  |  |
| **Yes** | 41 (44) | 25.8 (26.3) | |  | 26 | 25.5 |  | 18 | 27.7 |  | 11 | 12.9 |  | 30 | 40.5 |
| **No** | 91 (96) | 57.2 (57.5) | |  | 49 | 48 |  | 47 | 72.3 |  | 59 | 69.4 |  | 32 | 43.2 |
| **Missing value** | 27 (27) | 17.0 (16.2) | |  | 27 | 26.5 |  | 0 | 0 |  | 15 | 17.6 |  | 12 | 16.2 |
|  |  |  | |  |  |  |  |  |  |  |  |  |  |  |  |
| **Ever used a phone** |  |  | |  |  |  |  |  |  |  |  |  |  |  |  |
| **Yes** | 77 (82) | 48.4 (49.1) | |  | 36 | 35.3 |  | 46 | 70.8 |  | 30 | 35.3 |  | 47 | 63.5 |
| **No** | 55 (58) | 34.6 (34.7) | |  | 39 | 38.2 |  | 19 | 29.2 |  | 40 | 47.1 |  | 15 | 20.3 |
| **Missing value** | 27 (27) | 17.0 (16.2) | |  | 27 | 26.5 |  | 0 | 0 |  | 15 | 17.6 |  | 12 | 16.2 |
|  |  |  | |  |  |  |  |  |  |  |  |  |  |  |  |
| **Number of travel destinations** | 159 (167) | 2.4 (2.5) | |  | 102 | 2.0 |  | 65 | 3.3 |  | 85 | 1.8 |  | 74 | 3.1 |
|  |  |  | |  |  |  |  |  |  |  |  |  |  |  |  |
| **Travel time to health care center (hours)** | 131 (139) | 5.3 (5.2) | |  | 75 | 5.8 |  | 64 | 4.4 |  | 69 | 5.4 |  | 62 | 5.1 |
| **Do not know** | 1 (1) |  | |  | 0 |  |  | 1 |  |  | 1 |  |  | 0 |  |
| **Missing values** | 27 (27) |  | |  | 27 |  |  | 0 |  |  | 15 |  |  | 12 |  |
|  |  |  | |  |  |  |  |  |  |  |  |  |  |  |  |
| **Travel cost to a health care center ($)** | 121 (129) | 164.2 (167.5) | |  | 66 | 180.8 |  | 63 | 153.7 |  | 65 | 133.2 |  | 56 | 200.2 |
|  |  |  | |  |  |  |  |  |  |  |  |  |  |  |  |
| **Travel method to a health care center** |  |  | |  |  |  |  |  |  |  |  |  |  |  |  |
| **Car** | 71 (75) | 44.6 (44.9) | |  | 35 | 34.3 |  | 40 | 61.5 |  | 46 | 54.1 |  | 25 | 33.8 |
| **Other** | 61 (65) | 38.4 (38.9) | |  | 40 | 39.2 |  | 25 | 38.5 |  | 24 | 28.2 |  | 37 | 50.0 |
| **Missing value** | 27 (27) | 17.0 (16.2) | |  | 27 | 26.5 |  |  |  |  | 15 | 17.6 |  | 12 | 16.2 |
|  |  |  | |  |  |  |  |  |  |  |  |  |  |  |  |
| **Ever been unable to access a health care center** |  |  | |  |  |  |  |  |  |  |  |  |  |  |  |
| **Yes** | 95 (101) | 59.7 (60.5) | |  | 59 | 57.8 |  | 42 | 64.6 |  | 55 | 64.7 |  | 40 | 54.1 |
| **No** | 35 (37) | 22.0 (22.2) | |  | 15 | 14.7 |  | 22 | 33.8 |  | 14 | 16.5 |  | 21 | 28.4 |
| **Missing values** | 29 (29) | 18.3 (17.3) | |  | 28 | 27.5 |  | 1 | 1.5 |  | 16 | 18.8 |  | 13 | 17.6 |
|  |  |  | |  |  |  |  |  |  |  |  |  |  |  |  |
| **Reported at least one deceased child** |  |  | |  |  |  |  |  |  |  |  |  |  |  |  |
| **Yes** | 48 (50) | 30.2 (29.9) | |  | 30 | 29.4 |  | 20 | 30.8 |  | 32 | 37.6 |  | 16 | 21.6 |
| **No** | 108 (114) | 67.9 (68.3) | |  | 70 | 68.6 |  | 44 | 67.7 |  | 50 | 58.8 |  | 58 | 78.4 |
| **Missing values** | 3 (3) | 1.9 (1.8) | |  | 2 | 2.0 |  | 1 | 1.5 |  | 3 | 3.5 |  |  |  |
|  |  |  | |  |  |  |  |  |  |  |  |  |  |  |  |
| **HSV shedding** |  |  | |  |  |  |  |  |  |  |  |  |  |  |  |
| **Yes** | 28 (29) | 17.6 (17.4) | |  | 18 | 17.6 |  | 11 | 16.9 |  | 19 | 22.4 |  | 9 | 12.2 |
| **No** | 131 (138) | 82.4 (82.6) | |  | 84 | 82.4 |  | 54 | 83.1 |  | 66 | 77.6 |  | 65 | 87.8 |

a: Includes data from participants interviewed in 2015 and 2016. Only data collected in 2015 are displayed in these columns for the eight participants who were interviewed in both years.

b: The numbers in parentheses display the number of values, means, or percentages using the data from both 2015 and 2016 for the individuals interviewed in both years.

Among the 8 participants who were interviewed both in 2015 and 2016, mobile phone ownership was not static. Two participants who did not own phones in 2015 reported owning one in 2016, and one who reported owning a phone in 2015 no longer owned one in 2016.

Some participants reported that they may be able to travel to a health care center by car, including a government car, if necessary (53.8%, 71/132). 29.9% of all participants reported having at least 1 deceased child. More women than men reported having a deceased child (37.6% vs 21.6%), likely linked to women being the primary childcare providers in the population.

We aggregated HSV shedding across both HSV-1 and 2, and shedding almost certainly underestimates HSV prevalence. HSV is a virus that does not shed persistently and causes latent, asymptomatic infection; detecting 17.4% of HSV shedding in a population with data from a single time point from each participant indicates a relatively high prevalence of HSV infection in the population [6].

Table B: Description comparison of the characteristics of the participants and comparison by data collection year and gender^a^. Percentages and means are pooled estimates after applying Rubin rules (after multiple imputations).

|  | Recruited in 2015 and 2016 |  | Recruited in 2015 |  | Recruited in 2016 | p-value^b^ |  | Women |  | Men | p-value^c^ |
| --- | --- | --- | --- | --- | --- | --- | --- | --- | --- | --- | --- |
|  | **Percentage or mean** |  | **Percentage or mean** |  | **Percentage or mean** |  |  | **Percentage or mean** |  | **Percentage or mean** |  |
| Recruitment year |  |  |  |  |  |  |  |  |  |  |  |
| 2015 (Rainy season) | 64.2 |  |  |  |  |  |  | 62.4 |  | 66.2 | 0.612 |
| 2016 (Dry season) | 35.8 |  |  |  |  |  |  | 37.6 |  | 33.8 |  |
|  |  |  |  |  |  |  |  |  |  |  |  |
| Age group |  |  |  |  |  |  |  |  |  |  |  |
| 16-25 | 22.6 |  | 18.6 |  | 29.8 | 0.106 |  | 25.9 |  | 18.9 | 0.295 |
| 26-35 | 25.8 |  | 18.6 |  | 38.6 | 0.006 |  | 18.8 |  | 33.8 | 0.031 |
| 36-45 | 19.5 |  | 19.6 |  | 19.3 | 0.962 |  | 23.5 |  | 14.9 | 0.169 |
| 46-59 | 15.1 |  | 19.6 |  | 7.0 | **0.033** |  | 17.6 |  | 12.2 | 0.335 |
| 60+ | 17.0 |  | 23.5 |  | 5.3 | **0.003** |  | 14.1 |  | 20.3 | 0.303 |
|  |  |  |  |  |  |  |  |  |  |  |  |
| Gender |  |  |  |  |  |  |  |  |  |  |  |
| Women | 53.5 |  | 52.0 |  | 56.1 | 0.612 |  |  |  |  |  |
| Men | 46.5 |  | 48.0 |  | 43.9 |  |  |  |  |  |  |
|  |  |  |  |  |  |  |  |  |  |  |  |
| Mobile phone ownership |  |  |  |  |  |  |  |  |  |  |  |
| Yes | 28.2 |  | 29.3 |  | 26.3 | 0.710 |  | 14.3 |  | 44.2 | **<0.001** |
| No | 71.8 |  | 70.7 |  | 73.7 |  |  | 85.7 |  | 55.8 |  |
|  |  |  |  |  |  |  |  |  |  |  |  |
| Ever used a phone |  |  |  |  |  |  |  |  |  |  |  |
| Yes | 51.2 |  | 39.6 |  | 71.9 | **<0.001** |  | 36.7 |  | 67.8 | **<0.001** |
| No | 48.8 |  | 60.4 |  | 28.1 |  |  | 63.3 |  | 32.2 |  |
|  |  |  |  |  |  |  |  |  |  |  |  |
| Number of travel destinations | 2.4 |  | 2.0 |  | 3.1 | **<0.001** |  | 1.8 |  | 3.1 | **<0.001** |
|  |  |  |  |  |  |  |  |  |  |  |  |
| Travel time to health care center (hours) | 5.2 |  | 5.7 |  | 4.5 | 0.206 |  | 5.3 |  | 5.2 | 0.955 |
|  |  |  |  |  |  |  |  |  |  |  |  |
| Travel cost to a health care center ($) | 164.5 |  | 175.4 |  | 145.0 | 0.699 |  | 136.7 |  | 196.5 | 0.563 |
|  |  |  |  |  |  |  |  |  |  |  |  |
| Travel method to a health care center |  |  |  |  |  |  |  |  |  |  |  |
| Car | 53.9 |  | 48.8 |  | 63.2 | 0.104 |  | 64.9 |  | 41.4 | **0.008** |
| Other | 46.1 |  | 51.2 |  | 36.8 |  |  | 35.1 |  | 58.6 |  |
|  |  |  |  |  |  |  |  |  |  |  |  |
| Ever been unable to access a health care center |  |  |  |  |  |  |  |  |  |  |  |
| Yes | 73.9 |  | 79.3 |  | 64.3 | 0.057 |  | 80.7 |  | 66.1 | 0.076 |
| No | 26.1 |  | 20.7 |  | 35.7 |  |  | 19.3 |  | 33.9 |  |
|  |  |  |  |  |  |  |  |  |  |  |  |
| Reported at least one deceased child |  |  |  |  |  |  |  |  |  |  |  |
| Yes | 30.9 |  | 30.2 |  | 32.0 | 0.810 |  | 38.9 |  | 21.6 | **0.020** |
| No | 69.1 |  | 69.8 |  | 68.0 |  |  | 61.1 |  | 78.4 |  |
|  |  |  |  |  |  |  |  |  |  |  |  |
| HSV shedding |  |  |  |  |  |  |  |  |  |  |  |
| Yes | 17.6 |  | 17.6 |  | 17.5 | 0.987 |  | 22.4 |  | 12.2 | 0.092 |
| No | 82.4 |  | 82.4 |  | 82.5 |  |  | 77.6 |  | 87.8 |  |

a: Includes data collected in 2015 for the eight participants interviewed both years.

b: The p-value corresponds to tests comparing characteristics between recruitment years.

c: The p-value corresponds to tests comparing characteristics between genders.

Figure C: Visual comparison of the balance of the characteristics between mobile phone owners and non-phone owners. The Love plot provides a visual comparison of the covariate balance after multiple imputation with standardized difference between phone owners and non-phone owners. The value of the standardized difference indicates how imbalanced the covariate is: more frequent characteristics among mobile phone owners lead to standardized differences above 0. A filled circle indicates the difference in mean or proportion is statistically significant at p$\boldsymbol{\leq}$0.05, an open circle indicates a p>0.05, and the color indicates the gender when applicable (red for men, blue for women).

**Comparison stratified by gender and recruitment year**

Male mobile phone owners were significantly less likely to be 60+ years old, but no other significant differences were found between mobile phone owners and non-phone owners across gender strata (Figure D and Table C). In addition, the age and gender profile of mobile phone owners varied depending on the year of data collection. Mobile phone owners were significantly more likely to be aged 26-35 years and to be men in 2015 (rainy season) when compared with data collected in 2016 (dry season). Mobile phone owners also reported more travel destinations than non-phone owners in both 2015 and 2016, but the difference was not significant in 2015 (Figure D, and Table B).

Figure D: Love plot of the characterisrics of the participants stratified by recruitment year (A) and gender (B). Filled circles indicate that differences are statistically significant (p$\boldsymbol{\leq}$0.05) when comparing mobile phone owners to non-phone owners after estimating p-values using Rubin’s rules on the imputed data sets.

Table C: Values of the love plot comparing mobile phone owners to non-owners for the whole data set and after stratification on recruitment year and gender.

|  | **Standardized**  **difference** | **p-value^a^** |  | **Recruited in**  **2015** | |  | **Recruited in**  **2016** | |  | **Women** | |  | **Men** | |
| --- | --- | --- | --- | --- | --- | --- | --- | --- | --- | --- | --- | --- | --- | --- |
|  |  |  |  | **Standardized**  **difference** | **p-value^a^** |  | **Standardized**  **difference** | **p-value^a^** |  | **Standardized**  **difference** | **p-value^a^** |  | **Standardized**  **difference** | **p-value^a^** |
| **Age group** |  |  |  |  |  |  |  |  |  |  |  |  |  |  |
| **16-25** | 0.263 | 0.798 |  | 0.142 | 0.890 |  | 0.013 | 0.987 |  | -0.975 | 0.430 |  | 1.677 | 0.132 |
| **26-35** | 2.448 | **0.015** |  | 2.629 | **0.008** |  | 0.848 | 0.402 |  | 1.175 | 0.212 |  | 1.459 | 0.168 |
| **36-45** | -0.741 | 0.536 |  | -0.437 | 0.736 |  | -0.237 | 0.817 |  | -0.091 | 0.957 |  | -0.326 | 0.767 |
| **46-59** | -0.492 | 0.683 |  | -0.626 | 0.596 |  | -0.128 | 0.901 |  | 0.166 | 0.861 |  | -0.437 | 0.708 |
| **60+** | -2.699 | 0.058 |  | -2.322 | 0.088 |  | -2.091 | 0.201 |  | -0.811 | 0.542 |  | -3.053 | **0.028** |
|  |  |  |  |  |  |  |  |  |  |  |  |  |  |  |
| **Men** | 4.571 | **<0.001** |  | 4.068 | **0.001** |  | 1.985 | 0.058 |  |  |  |  |  |  |
|  |  |  |  |  |  |  |  |  |  |  |  |  |  |  |
| **Number of adults in household** | 0.904 | 0.376 |  | 0.970 | 0.343 |  | -0.082 | 0.936 |  | 0.642 | 0.526 |  | 1.666 | 0.160 |
|  |  |  |  |  |  |  |  |  |  |  |  |  |  |  |
| **HSV shedding**  **detected** | -2.0 | 0.113 |  | -2.038 | 0.121 |  | -0.034 | 0.973 |  | -0.203 | 0.856 |  | -1.766 | 0.148 |
|  |  |  |  |  |  |  |  |  |  |  |  |  |  |  |
| **Reported at least one deceased child** | -0.354 | 0.746 |  | 0.019 | 0.982 |  | 0.219 | 0.826 |  | 0.727 | 0.504 |  | -0.055 | 0.962 |
|  |  |  |  |  |  |  |  |  |  |  |  |  |  |  |
| **Travel time to a health care center** | -2.474 | **0.032** |  | -2.611 | **0.035** |  | -0.873 | 0.383 |  | -0.502 | 0.645 |  | -2.451 | **0.031** |
|  |  |  |  |  |  |  |  |  |  |  |  |  |  |  |
| **Able to travel to a health care center with a car** | -0.172 | 0.868 |  | -0.131 | 0.909 |  | -0.606 | 0.539 |  | -0.566 | 0.572 |  | 1.551 | 0.165 |
|  |  |  |  |  |  |  |  |  |  |  |  |  |  |  |
| **Unable to access a health care center** | -0.817 | 0.455 |  | -0.405 | 0.703 |  | -1.581 | 0.107 |  | 1.172 | 0.382 |  | -0.927 | 0.409 |
|  |  |  |  |  |  |  |  |  |  |  |  |  |  |  |
| **Number of travel destinations** | 3.539 | **0.002** |  | 3.179 | 0.**0.018** |  | 2.492 | **0.013** |  | 2.447 | **0.040** |  | 0.945 | 0.412 |

a: The p-value corresponds to testing the null hypothesis that there is no unstandardized difference between mobile phone owners and non-phone owners in the corresponding strata after applying Rubin’s rules to the imputed data sets.

Table D: Comparison of the characteristics of phone owners and non-phone owners^ab^.

|  | **Mobile phone owners** | **Non-phone owners** | **p-value** |  | **Women** | | |  | **Men** | | |
| --- | --- | --- | --- | --- | --- | --- | --- | --- | --- | --- | --- |
|  |  |  |  |  | **Mobile phone owners** | **Non-phone owners** | **p-value** |  | **Mobile phone owners** | **Non-phone owners** | **p-value** |
|  | **Percentage (%) or mean** | **Percentage (%) or mean** |  |  | **Percentage (%) or mean** | **Percentage (%) or mean** |  |  | **Percentage (%) or mean** | **Percentage (%) or mean** |  |
| **Age group** |  |  |  |  |  |  |  |  |  |  |  |
| **16-25 years** | 23.6 | 21.6 | 0.798 |  | 16.6 | 27.4 | 0.430 |  | 26.3 | 11.2 | 0.132 |
| **26-35 years** | 40.1 | 20.3 | **0.015** |  | 32.8 | 16.6 | 0.212 |  | 43.0 | 27.0 | 0.168 |
| **36-45 years** | 16.1 | 20.8 | 0.536 |  | 22.7 | 23.5 | 0.957 |  | 13.5 | 16.1 | 0.767 |
| **46-59 years** | 13.2 | 16.1 | 0.683 |  | 19.4 | 17.2 | 0.861 |  | 10.7 | 14.0 | 0.708 |
| **60+ years** | 7.0 | 21.2 | 0.058 |  | 8.5 | 15.3 | 0.542 |  | 6.6 | 31.8 | **0.028** |
|  |  |  |  |  |  |  |  |  |  |  |  |
| **Men** | 72.0 | 35.9 | **<0.001** |  |  |  |  |  |  |  |  |
|  |  |  |  |  |  |  |  |  |  |  |  |
| **Number of adults in the household** | 2.1 | 1.8 | 0.376 |  | 2.5 | 1.9 | 0.526 |  | 1.9 | 1.6 | 0.160 |
|  |  |  |  |  |  |  |  |  |  |  |  |
| **HSV shedding** | 9.4 | 20.6 | 0.113 |  | 19.9 | 22.3 | 0.856 |  | 5.2 | 17.5 | 0.148 |
|  |  |  |  |  |  |  |  |  |  |  |  |
| **Reported at least one deceased child** | 28.6 | 31.3 | 0.746 |  | 47.3 | 36.7 | 0.504 |  | 21.2 | 21.7 | 0.962 |
|  |  |  |  |  |  |  |  |  |  |  |  |
| **Travel time to a health care center** | 3.8 | 5.7 | **0.032** |  | 4.7 | 5.4 | 0.645 |  | 3.5 | 6.4 | 0**.031** |
|  |  |  |  |  |  |  |  |  |  |  |  |
| **Able to travel to a health care center with a car** | 53.5 | 55.0 | 0.868 |  | 58.3 | 66.7 | 0.572 |  | 51.6 | 34.2 | 0.165 |
|  |  |  |  |  |  |  |  |  |  |  |  |
| **Unable to access a health care center** | 69.4 | 75.8 | 0.455 |  | 89.0 | 78.0 | 0.382 |  | 61.8 | 71.8 | 0.409 |
|  |  |  |  |  |  |  |  |  |  |  |  |
| **Number of travel destinations** | 3.1 | 2.1 | **0.002** |  | 2.7 | 1.6 | **0.040** |  | 3.3 | 2.9 | 0.412 |

a: Only the data from 2015 are used for the eight participants interviewed in both years.

b: Percentages and means were estimated after applying Rubin’s rules to the imputed data sets.

**Model selection for the logistic regression analysis**

We used logistic regression analysis to investigate the relationships between mobile phone ownership and individual behaviors and characteristics. We fit a multivariate logistic regression model to each imputed data set and applied Rubin’s rules to pool the estimates. We explored models including various combinations of predictors (Table E).

Table E: Covariates included in the models screened during the logistic regression analysis^a^.

| **Potential predictors** | **Model 1** | **Model 2** | **Model 3** | **Model 4** | **Model 5** | **Model 6** | **Model 7** | **Model 8** | **Model 9** | **Model 10** | **Model 11** | **Model 12** | **Model 13** | **Model 14** |
| --- | --- | --- | --- | --- | --- | --- | --- | --- | --- | --- | --- | --- | --- | --- |
| Age |  |  |  |  |  |  |  |  |  |  |  |  |  |  |
| Gender |  |  |  |  |  |  |  |  |  |  |  |  |  |  |
| Unable to access a health care center |  |  |  |  |  |  |  |  |  |  |  |  |  |  |
| Number of adults in the household |  |  |  |  |  |  |  |  |  |  |  |  |  |  |
| HSV shedding |  |  |  |  |  |  |  |  |  |  |  |  |  |  |
| Recruitment year |  |  |  |  |  |  |  |  |  |  |  |  |  |  |
| Travel time to a health care center |  |  |  |  |  |  |  |  |  |  |  |  |  |  |
| Quadratic term for travel time to a health care center |  |  |  |  |  |  |  |  |  |  |  |  |  |  |
| Number of travel destinations |  |  |  |  |  |  |  |  |  |  |  |  |  |  |
| Quadratic term for number of travel destinations |  |  |  |  |  |  |  |  |  |  |  |  |  |  |
| Able to travel to a health care center with a car |  |  |  |  |  |  |  |  |  |  |  |  |  |  |
| Reported at least 1 deceased child |  |  |  |  |  |  |  |  |  |  |  |  |  |  |
| Interaction term between travel time to a health care center and gender |  |  |  |  |  |  |  |  |  |  |  |  |  |  |
| Interaction term between number of travel destinations and gender |  |  |  |  |  |  |  |  |  |  |  |  |  |  |

a: The cell is green if a potential predictor was included in a specific model, it is empty if it was not.

We performed 15 imputations, and with each imputed dataset we calculated the average agreement for the predicted outcome, in this case mobile phone ownership, by jackknife. We then calculated the average agreement over all the imputations for each model (Table F). We repeated the procedure after excluding the 27 participants with missing values for the outcome as a sensitivity analysis (25 who were interviewed during the pilot phase and 2 who presented as guardians of children and did not complete interviews) (Table G). The final model was chosen by selecting the model maximizing the average agreement estimated by jackknife. The agreement was calculated over all the imputations and averaged.

Table F: Agreement of the models when fit to all participants (N=159).

| **Model** | **Agreement** |
| --- | --- |
| Model 3 | 0.746751 |
| Model 11 | 0.745073 |
| Model 5 | 0.744654 |
| Model 1 | 0.744235 |
| Model 13 | 0.739623 |
| Model 14 | 0.738365 |
| Model 12 | 0.732075 |
| Model 7 | 0.701048 |
| Model 6 | 0.69979 |
| Model 4 | 0.698952 |
| Model 8 | 0.695597 |
| Model 10 | 0.693082 |
| Model 2 | 0.691405 |
| Model 9 | 0.689308 |

Table G: Agreement of the models when fit to participants who provided phone ownership data (N=132).

| **Model** | **Agreement** |
| --- | --- |
| Model 5 | 0.723232 |
| Model 14 | 0.722727 |
| Model 12 | 0.715657 |
| Model 3 | 0.710606 |
| Model 11 | 0.707071 |
| Model 1 | 0.706061 |
| Model 4 | 0.69596 |
| Model 13 | 0.69596 |
| Model 6 | 0.690404 |
| Model 8 | 0.688384 |
| Model 10 | 0.687879 |
| Model 2 | 0.678283 |
| Model 7 | 0.665152 |
| Model 9 | 0.644949 |

The best performing model after excluding the participants with missing values for mobile phone ownership was Model 5 (Table G). It also showed agreement values that were very similar to the best performing model that included the participants with missing values for mobile phone ownership (Table F). We then used Model 5 as the final model. The main associations of Model 5 are presented in the manuscript and the details of all the associations are presented in Table H.

This confirmed that phone owners were more often men than women overall (adjusted odd ratios (aOR)=3.8*, CI95%:1.4-10.4) and during 2015 (aOR=4.9*, CI95%: 1.1-20.7). However, we did not detect an association between phone ownership and men from data collected in 2016 only. Across the full dataset, we found that phone owners were less frequently aged over 60 years (aOR=0.2, CI95%:0-1.1), especially among men (aOR=0*, CI95%: 0-0.6). Phone ownership among women overall was too low to detect age patterns.

Phone owners also reported a greater number of travel destinations in the 12 months preceding the interview, (aOR=1.3, CI95%: 0.9-1.8) as well as shorter travel times to a health care center (aOR=0.9, CI95%:0.8-1.0), although these relationships were not statistically significant (Table G). When data were stratified by collection year, we observed that phone owners reported more travel destinations than non-phone owners and shorter travel times to health care centers in both 2015 (rainy season) and 2016 (dry season), though these were not statistically significant.

Table H: Covariates associated with mobile phone ownership. The strength of the association is estimated by adjusted odds ratios (aOR) by logistic regression in the global data set, and stratified by data collection year, and gender.

|  | **Recruited in 2015 and 2016^a^**  **(N=159)** | | | | |  | **Recruited in**  **2015**  **(N=102)** | | | | |  | **Recruited in**  **2016**  **(N=65)** | | | | |  | **Women**  **(N=85)** | | | | |  | **Men**  **(N=74)** | | | | |
| --- | --- | --- | --- | --- | --- | --- | --- | --- | --- | --- | --- | --- | --- | --- | --- | --- | --- | --- | --- | --- | --- | --- | --- | --- | --- | --- | --- | --- | --- |
|  | **aOR** |  | **95% CI** |  | **p-value** |  | **aOR** |  | **95% CI** |  | **p-value** |  | **aOR** |  | **95% CI** |  | **p-value** |  | **aOR** |  | **95% CI** |  | **p-value** |  | **aOR** |  | **95% CI** |  | **p-value** |
| **Recruitment year** |  |  |  |  |  |  |  |  |  |  |  |  |  |  |  |  |  |  |  |  |  |  |  |  |  |  |  |  |  |
| **2015 (Rainy season)** | 1 |  |  |  |  |  |  |  |  |  |  |  |  |  |  |  |  |  | 1 |  |  |  |  |  | 1 |  |  |  |  |
| **2016 (Dry season)** | 0.3 |  | 0.1-0.9 |  | **0.025** |  |  |  |  |  |  |  |  |  |  |  |  |  | 0.5 |  | 0.1-2.8 |  | 0.461 |  | 0.1 |  | 0-0.7 |  | **0.017** |
|  |  |  |  |  |  |  |  |  |  |  |  |  |  |  |  |  |  |  |  |  |  |  |  |  |  |  |  |  |  |
| **Age group** |  |  |  |  |  |  |  |  |  |  |  |  |  |  |  |  |  |  |  |  |  |  |  |  |  |  |  |  |  |
| **16-25** | 1 |  |  |  |  |  | 1 |  |  |  |  |  | 1 |  |  |  |  |  | 1 |  |  |  |  |  | 1 |  |  |  |  |
| **26-35** | 1.3 |  | 0.4-4 |  | 0.640 |  | 2.2 |  | 0.4-12.3 |  | 0.377 |  | 0.9 |  | 0.2-4.1 |  | 0.913 |  | 2.4 |  | 0.3-18.1 |  | 0.398 |  | 0.6 |  | 0.1-4.0 |  | 0.564 |
| **36-45** | 0.6 |  | 0.1-2.4 |  | 0.446 |  | 0.8 |  | 0.1-5.9 |  | 0.805 |  | 0.8 |  | 0.1-5.2 |  | 0.814 |  | 1.2 |  | 0.1-13.3 |  | 0.882 |  | 0.2 |  | 0-2.1 |  | 0.173 |
| **46-59** | 0.4 |  | 0.1-2.1 |  | 0.270 |  | 0.4 |  | 0-3.7 |  | 0.412 |  | 1.1 |  | 0.1-21.8 |  | 0.938 |  | 1.0 |  | 0-17.2 |  | 0.987 |  | 0.1 |  | 0-1.4 |  | 0.088 |
| **>=60** | 0.2 |  | 0-1.1 |  | 0.061 |  | 0.2 |  | 0-1.8 |  | 0.153 |  | 0 |  | 0-Inf |  | 0.994 |  | 0.5 |  | 0-11.1 |  | 0.668 |  | 0 |  | 0-0.6 |  | **0.022** |
|  |  |  |  |  |  |  |  |  |  |  |  |  |  |  |  |  |  |  |  |  |  |  |  |  |  |  |  |  |  |
| **Gender** |  |  |  |  |  |  |  |  |  |  |  |  |  |  |  |  |  |  |  |  |  |  |  |  |  |  |  |  |  |
| **Women** | 1 |  |  |  |  |  | 1 |  |  |  |  |  | 1 |  |  |  |  |  |  |  |  |  |  |  |  |  |  |  |  |
| **Men** | 3.8 |  | 1.4-10.4 |  | **0.009** |  | 4.9 |  | 1.1-20.7 |  | **0.032** |  | 1.4 |  | 0.2-7.8 |  | 0.717 |  |  |  | - |  |  |  |  |  | - |  |  |
|  |  |  |  |  |  |  |  |  |  |  |  |  |  |  |  |  |  |  |  |  |  |  |  |  |  |  |  |  |  |
| **Number of adults in the household** | 1.4 |  | 0.9-2.1 |  | 0.142 |  | 1.4 |  | 0.8-2.6 |  | 0.226 |  | 0.9 |  | 0.4-2.1 |  | 0.836 |  | 1.2 |  | 0.8-1.6 |  | 0.415 |  | 3.2 |  | 0.8-11.9 |  | 0.086 |
|  |  |  |  |  |  |  |  |  |  |  |  |  |  |  |  |  |  |  |  |  |  |  |  |  |  |  |  |  |  |
| **Number of travel destinations** | 1.3 |  | 0.9-1.8 |  | 0.105 |  | 1.3 |  | 0.8-2.1 |  | 0.217 |  | 1.4 |  | 0.8-2.4 |  | 0.291 |  | 1.8 |  | 0.9-3.5 |  | 0.088 |  | 1.2 |  | 0.7-1.9 |  | 0.455 |
|  |  |  |  |  |  |  |  |  |  |  |  |  |  |  |  |  |  |  |  |  |  |  |  |  |  |  |  |  |  |
| **Travel time to a health care center** | 0.9 |  | 0.8-1 |  | 0.118 |  | 0.9 |  | 0.7-1.0 |  | 0.139 |  | 1.0 |  | 0.8-1.1 |  | 0.626 |  | 0.9 |  | 0.7-1.1 |  | 0.205 |  | 0.9 |  | 0.7-1.1 |  | 0.210 |
|  |  |  |  |  |  |  |  |  |  |  |  |  |  |  |  |  |  |  |  |  |  |  |  |  |  |  |  |  |  |
| **Ever been unable to access**  **health care center** |  |  |  |  |  |  |  |  |  |  |  |  |  |  |  |  |  |  |  |  |  |  |  |  |  |  |  |  |  |
| **No** | 1 |  |  |  |  |  | 1 |  |  |  |  |  | 1 |  |  |  |  |  | 1 |  |  |  |  |  | 1 |  |  |  |  |
| **Yes** | 1.0 |  | 0.4-2.8 |  | 0.994 |  | 1.0 |  | 0.2-4.5 |  | 0.966 |  | 0.5 |  | 0.1-1.9 |  | 0.270 |  | 2.1 |  | 0.2-19.6 |  | 0.522 |  | 0.6 |  | 0.1-2.9 |  | 0.546 |
|  |  |  |  |  |  |  |  |  |  |  |  |  |  |  |  |  |  |  |  |  |  |  |  |  |  |  |  |  |  |
| **Travel method to a health care center** |  |  |  |  |  |  |  |  |  |  |  |  |  |  |  |  |  |  |  |  |  |  |  |  |  |  |  |  |  |
| **Other** | 1 |  |  |  |  |  | 1 |  |  |  |  |  | 1 |  |  |  |  |  | 1 |  |  |  |  |  | 1 |  |  |  |  |
| **Car** | 0.9 |  | 0.3-2.7 |  | 0.915 |  | 0.8 |  | 0.1-4.2 |  | 0.771 |  | 1.1 |  | 0.2-5.1 |  | 0.918 |  | 0.5 |  | 0.1-3.6 |  | 0.455 |  | 1.5 |  | 0.4-6.5 |  | 0.553 |

a: Includes data from participants interviewed in 2015 and 2016. For the eight participants who were interviewed in both years, only the data collected in 2015 is included in these columns.

**Difference in means after propensity score matching**

We used propensity score matching to minimize confounding biases. We assessed the difference in the travel time to a health care center between mobile phone owners and non-phone owners. Estimates of the mean travel time to a health care center were lower among phone owners compared to non-phone owners (Table I). The confidence interval (CI) of the pooled estimate of the mean difference in travel time to a health care center between phone owners and non-phone owners was large, approximately -2.6 hours, and close to statistical significance (95%CI: -5.5-0.3) (Table H).

Figure E: Mean reduction of time to travel to a health care center after matching on propensity score using data collected in 2015, or in 2016, or both but assumed independent for the 8 participants interviewed twice. Open circles indicate that the 95% confidence interval includes 0.

Table I: Detailed values of the mean reduction in travel time to a health care center for phone owners compared to non-phone owners after matching on propensity score

| **Stratum** | **Mean reduction in travel time to a health care center (hours)** | **95% CI** |
| --- | --- | --- |
| All data | -2.63 | -5.54-0.28 |
| Men | -2.53 | -8.19-3.14 |
| Women | -0.88 | -5.41-3.65 |
| Rainy season - 2015 | -1.41 | -5.88-3.07 |
| Dry season - 2016 | -1.17 | -4.76-2.41 |

**Sensitivity analysis on the mean reduction of the travel time to a health care center**

We investigated the potential impact of using one value rather than another for the 8 participants interviewed both in 2015 and 2016 on the mean reduction of travel time to a health care center. Figure E and Table J show that picking one value rather than another has little impact on the estimates. We used the data chronologically collected first (2015) in the main analyses.

Table J: Detailed values of the mean reduction of the travel time to a health care center after matching on propensity score using data collected in 2015, or in 2016, or both but assumed independent for the 8 participants interviewed twice.

| **Alternatives to manage data from participants interviewed twice** | **Mean reduction of the travel time to a health care center (hours)** | **95% CI** |
| --- | --- | --- |
| 2015 values for participants interviewed twice | -2.63 | -5.54-0.28 |
| 2016 values for participants interviewed twice | -2.03 | -4.63-0.57 |
| 2015 and 2016 values for participants interviewed twice | -2.23 | -5.03-0.57 |

**Reconstructed contact network**

We reconstructed a contact network by recruitment year and included all participants using self-reported sexual contacts describing the six months preceding the survey as well as family/partner ties. All named sexual partners were not participants in the study, but they appear as additional vertices and provide a more complete picture of the connectivity throughout the population. The edges in the reconstructed network reflect sexual contacts or family/partners ties. We completed a descriptive analysis of the networks focusing on degree distribution.

Table K: Description of the number of edges among mobile phone owners and non-phone owners in the whole data set and by gender and recruitment year strata.

|  | **Mean number of edges** | **Median number of edges** | **IQR** | **p-value** |
| --- | --- | --- | --- | --- |
| **All data** |  |  |  |  |
| **Non-phone owners** | 1.8 | 2 | 1-2 | 0.025 |
| **Phone owners** | 2.66 | 2 | 1-3 |  |
|  |  |  |  |  |
| **Recruitment year 2015 – Rainy season** |  |  |  |  |
| **Non-phone owners** | 1.55 | 1 | 1-2 | 0.082 |
| **Phone owners** | 2.19 | 2 | 1-3 |  |
|  |  |  |  |  |
| **Recruitment year 2016 – Dry season** |  |  |  |  |
| **Non-phone owners** | 2.06 | 2 | 1-2.5 | 2.06 |
| **Phone owners** | 3.33 | 2 | 2-4.75 | 3.33 |
|  |  |  |  |  |
| **Women** |  |  |  |  |
| **Non-phone owners** | 1.73 | 2 | 1-2 | 1.73 |
| **Phone owners** | 2.42 | 2 | 1-3 | 2.42 |
|  |  |  |  |  |
| **Men** |  |  |  |  |
| **Non-phone owners** | 1.92 | 1.5 | 1-2 | 1.92 |
| **Phone owners** | 2.75 | 2 | 1-3.25 | 2.75 |

We found weak evidence that mobile phone owners had a greater number of contacts, or edges, when compared to non-phone owners (Table K and Figure F). Phone owners reported 2.2 edges on average vs non-phone owners, who reported 1.6 edges on average (p=0.082) in 2015. In 2016, phone owners reported 3.3 edges on average and non-phone owners reported 2.1 edges on average (p=0.082). In 2015, phone owners vs non-phone owners reported a median of 2 (interquartile range (IQR): 1-3) vs 1 (IQR: 1-2.5) edges respectively. In 2016, both groups reported a median of 2 edges (IQR: 1-2.5 and 2-4.75 respectively).

Figure F: Distribution of the number of edges in the reconstructed networks broken down by phone ownership for the participants, children, and named contacts who were not participants in the study (A) and participants only (B).

**Additional estimates of biases**

In addition to the analysis presented above, we estimated biases in data representativeness among phone users by calculating the ratio of the mean value for quantitative variables or the ratio of the proportion for categorical variables or ordered variables in phone owners over the same estimates among all participants as well as among non-phone owners. We calculated the 95% CI of those ratios after making single imputations embedded in bootstrap (1000 samples) with the percentile method [7]. We stratified the analysis by gender and by data collection year.

The number of travel destinations in the 12 months preceding the survey was greater among phone owners compared to all participants (ratio of 1.3*, 95%CI: 1.0-1.6) and when comparing phone owners to non-phone owners (ratio 1.5*, CI95%: 1.0-2.0) (Table M).

When using data collected exclusively from phone owners to represent the entire population, the estimate of the mean travel time to a health care center (ratio=0.7*, 95%CI: 0.5-1.0) was underestimated by 1.6 hours (95%CI: -3.2—0.2). When stratifying by gender, a bias of shorter travel times to health care centers was observed in men but not in women. Women make up 26.8% (11/41) of phone owners, and among them, phone owners had a ratio for mean travel time to a health care center of 0.9 (CI95%:0.5-1.3) while in men, this ratio was 0.7* (CI95%: 0.5-0.9) (Table N).

We observed a similar pattern, though more pronounced, when using non-phone owners as the reference group. This also held true for lower (25^th^ percentile) and upper (75^th^ percentile) values of travel times to a health care center among men. Compared to all male study participants, male phone owners reported shorter mean travel times to a health care center by 1.7 hours, with 0.4 and 2.8 fewer travel hours for the 25^th^ and 75^th^ percentile values, respectively. When comparing male phone owners to male non-phone owners, mean travel times to a health care center were 3.1 hours shorter (95%CI: -6.2—0.5) and for the 25^th^ and 75^th^ percentile values, travel times to health care were 1 hour shorter (95%CI; -2.5-0) and 5.4 hours shorter (95%CI: -9.0-0), respectively (Table M).

Table L: Distribution of the recent travel destinations among mobile phone owners and non-phone owners.

| Mobile phone network reception | Village | Visits from mobile phone owners  (N=186) | | Visits from non-  phone owners  (N=325) | |
| --- | --- | --- | --- | --- | --- |
|  |  | **N** | **%** | **N** | **%** |
| Easily available | Etengwa | 21 | 11.3 | 34 | 10.5 |
|  | Opuwo | 15 | 8.1 | 17 | 5.2 |
|  | Okanguati | 13 | 7.0 | 24 | 7.4 |
|  | Okahua | 8 | 4.3 | 18 | 5.5 |
|  | Omuhonga | 6 | 3.2 | 6 | 1.8 |
|  | Etanga | 5 | 2.7 | 11 | 3.4 |
|  | Otjihende | 5 | 2.7 | 13 | 4.0 |
|  | Okakora | 4 | 2.2 | 3 | 0.9 |
|  | Oromukandi | 3 | 1.6 | 8 | 2.5 |
|  | Otjituka | 2 | 1.1 | 3 | 0.9 |
|  | Walvis Bay | 2 | 1.1 | 0 | 0.0 |
|  | Likokola | 1 | 0.5 | 0 | 0.0 |
|  | Ohondungu | 1 | 0.5 | 2 | 0.6 |
|  | Ohungumure | 1 | 0.5 | 0 | 0.0 |
|  | Okanyandi | 1 | 0.5 | 1 | 0.3 |
|  | Okapara | 1 | 0.5 | 3 | 0.9 |
|  | Okapawe | 1 | 0.5 | 0 | 0.0 |
|  | Ombutisauri | 1 | 0.5 | 2 | 0.6 |
|  | Otjinungwa | 1 | 0.5 | 4 | 1.2 |
|  | Orombamba | 0 | 0.0 | 2 | 0.6 |
|  | Okahama | 0 | 0.0 | 1 | 0.3 |
|  | Okakondorokwa | 0 | 0.0 | 1 | 0.3 |
|  | Omungwiti Wakekoro | 0 | 0.0 | 1 | 0.3 |
|  | Omuramba | 0 | 0.0 | 1 | 0.3 |
|  | Ongondjonambari | 0 | 0.0 | 1 | 0.3 |
|  | Windhoek | 0 | 0.0 | 1 | 0.3 |
| Limited | Otjitanda | 17 | 9.1 | 43 | 13.2 |
|  | Embwende | 7 | 3.8 | 11 | 3.4 |
|  | Omuatjivingo | 4 | 2.2 | 17 | 5.2 |
|  | Otjikoyo | 2 | 1.1 | 2 | 0.6 |
|  | Otutati | 2 | 1.1 | 1 | 0.3 |
|  | Ozowonduombe | 2 | 1.1 | 0 | 0.0 |
|  | Ombuyuwandume | 1 | 0.5 | 3 | 0.9 |
|  | Orokatuwo | 1 | 0.5 | 4 | 1.2 |
|  | Ozija | 1 | 0.5 | 8 | 2.5 |
|  | Orukaue | 0 | 0.0 | 1 | 0.3 |
|  | Ozohorongo | 0 | 0.0 | 1 | 0.3 |
| None | Ombepera | 1 | 0.5 | 2 | 0.6 |
|  | Otjizu | 1 | 0.5 | 2 | 0.6 |
|  | Roidrum | 1 | 0.5 | 0 | 0.0 |
|  | A different country | 0 | 0.0 | 3 | 0.9 |
|  | Otjandjeu | 0 | 0.0 | 1 | 0.3 |
| Unlikely but not confirmed | Onyezu | 4 | 2.2 | 3 | 0.9 |
|  | Epupa | 3 | 1.6 | 0 | 0.0 |
|  | Otjandawe | 3 | 1.6 | 3 | 0.9 |
|  | Epembe | 2 | 1.1 | 0 | 0.0 |
|  | Oruseu | 2 | 1.1 | 0 | 0.0 |
|  | Osana | 2 | 1.1 | 0 | 0.0 |
|  | Oshakati | 2 | 1.1 | 1 | 0.3 |
|  | Owonduombe | 2 | 1.1 | 0 | 0.0 |
|  | Amande | 1 | 0.5 | 0 | 0.0 |
|  | Ehomba | 1 | 0.5 | 0 | 0.0 |
|  | Ejao | 1 | 0.5 | 0 | 0.0 |
|  | Ekambu | 1 | 0.5 | 0 | 0.0 |
|  | Manta | 1 | 0.5 | 0 | 0.0 |
|  | Ngombuyondumwe | 1 | 0.5 | 0 | 0.0 |
|  | Oakatje | 1 | 0.5 | 0 | 0.0 |
|  | Okahozu | 1 | 0.5 | 0 | 0.0 |
|  | Okamanga | 1 | 0.5 | 1 | 0.3 |
|  | Okapaizirwa | 1 | 0.5 | 1 | 0.3 |
|  | Okavandje | 1 | 0.5 | 0 | 0.0 |
|  | Okouwore | 1 | 0.5 | 0 | 0.0 |
|  | Olurumbu | 1 | 0.5 | 0 | 0.0 |
|  | Ombiyindjandu | 1 | 0.5 | 0 | 0.0 |
|  | Ombwidjawandi | 1 | 0.5 | 0 | 0.0 |
|  | Omiramba | 1 | 0.5 | 1 | 0.3 |
|  | Omokukaze | 1 | 0.5 | 0 | 0.0 |
|  | Omurambaondueze | 1 | 0.5 | 0 | 0.0 |
|  | Ondamayavirera | 1 | 0.5 | 0 | 0.0 |
|  | Onyuva | 1 | 0.5 | 0 | 0.0 |
|  | Oruvaendjai | 1 | 0.5 | 0 | 0.0 |
|  | Otazuma | 1 | 0.5 | 0 | 0.0 |
|  | Otjaharavara | 1 | 0.5 | 0 | 0.0 |
|  | Otjihunwa | 1 | 0.5 | 0 | 0.0 |
|  | Otjikunda | 1 | 0.5 | 0 | 0.0 |
|  | Otjimborombonga | 1 | 0.5 | 1 | 0.3 |
|  | Otjindave | 1 | 0.5 | 0 | 0.0 |
|  | Otjinduu | 1 | 0.5 | 0 | 0.0 |
|  | Otjivero | 1 | 0.5 | 1 | 0.3 |
|  | Otjozongombe | 1 | 0.5 | 0 | 0.0 |
|  | Otuani | 1 | 0.5 | 0 | 0.0 |
|  | Otuhana | 1 | 0.5 | 0 | 0.0 |
|  | Ovaarandara | 1 | 0.5 | 0 | 0.0 |
|  | Ovingeja | 1 | 0.5 | 0 | 0.0 |
|  | Ekarwondjiwo | 0 | 0.0 | 3 | 0.9 |
|  | Ombangona | 0 | 0.0 | 3 | 0.9 |
|  | Okakuyu | 0 | 0.0 | 2 | 0.6 |
|  | Okapembei | 0 | 0.0 | 2 | 0.6 |
|  | Otjerunda | 0 | 0.0 | 2 | 0.6 |
|  | Otjiparara | 0 | 0.0 | 2 | 0.6 |
|  | Otjipemba | 0 | 0.0 | 2 | 0.6 |
|  | Ovikotorongo | 0 | 0.0 | 2 | 0.6 |
|  | Ovipereke | 0 | 0.0 | 2 | 0.6 |
|  | Karokatodo | 0 | 0.0 | 1 | 0.3 |
|  | Komuyendangoro | 0 | 0.0 | 1 | 0.3 |
|  | Ojiza | 0 | 0.0 | 1 | 0.3 |
|  | Okaheza | 0 | 0.0 | 1 | 0.3 |
|  | Okakaua | 0 | 0.0 | 1 | 0.3 |
|  | Okarukoro | 0 | 0.0 | 1 | 0.3 |
|  | Okaute | 0 | 0.0 | 1 | 0.3 |
|  | Omahunga | 0 | 0.0 | 1 | 0.3 |
|  | Omande | 0 | 0.0 | 1 | 0.3 |
|  | Omasitu | 0 | 0.0 | 1 | 0.3 |
|  | Ombazu | 0 | 0.0 | 1 | 0.3 |
|  | Ombinjuandu | 0 | 0.0 | 1 | 0.3 |
|  | Ombuyandwe | 0 | 0.0 | 1 | 0.3 |
|  | Ombwhonga | 0 | 0.0 | 1 | 0.3 |
|  | Omiheke | 0 | 0.0 | 1 | 0.3 |
|  | Omimire | 0 | 0.0 | 1 | 0.3 |
|  | Omirora | 0 | 0.0 | 1 | 0.3 |
|  | Omundjaondu | 0 | 0.0 | 1 | 0.3 |
|  | Omungundiuakekuro | 0 | 0.0 | 1 | 0.3 |
|  | Omwangete | 0 | 0.0 | 1 | 0.3 |
|  | Ondendu | 0 | 0.0 | 1 | 0.3 |
|  | Ondeni | 0 | 0.0 | 1 | 0.3 |
|  | Ondunta Kapapi | 0 | 0.0 | 1 | 0.3 |
|  | Orokazema | 0 | 0.0 | 1 | 0.3 |
|  | Oroseu | 0 | 0.0 | 1 | 0.3 |
|  | Oruhungu | 0 | 0.0 | 1 | 0.3 |
|  | Orujombo | 0 | 0.0 | 1 | 0.3 |
|  | Orumbororo | 0 | 0.0 | 1 | 0.3 |
|  | Otjavaiya | 0 | 0.0 | 1 | 0.3 |
|  | Oviana | 0 | 0.0 | 1 | 0.3 |
|  | Ovijere | 0 | 0.0 | 1 | 0.3 |
|  | Ovindjete | 0 | 0.0 | 1 | 0.3 |
|  | Owamuve | 0 | 0.0 | 1 | 0.3 |
|  | Owareva | 0 | 0.0 | 1 | 0.3 |
|  | Ozana | 0 | 0.0 | 1 | 0.3 |
|  | Ozonduwombe | 0 | 0.0 | 1 | 0.3 |
|  | Ozosene | 0 | 0.0 | 1 | 0.3 |

Table M: Ratio of the estimates based on mobile phone owners with reference to all participants or non-phone owners.

|  | **Reference: all participants** | | | | | | | |  | **Reference: non-phone owners** | | | | | | | |
| --- | --- | --- | --- | --- | --- | --- | --- | --- | --- | --- | --- | --- | --- | --- | --- | --- | --- |
|  | **Ratio** | **95% CI** |  | **Women** | |  | **Men** | |  | **Ratio** | **95% CI** |  | **Women** | |  | **Men** | |
|  |  |  |  | **Ratio** | **95% CI** |  | **Ratio** | **95% CI** |  |  |  |  | **Ratio** | **95% CI** |  | **Ratio** | **95% CI** |
| **Mean number of people living in the household** | 1.076 | 0.908-1.270 |  |  |  |  |  |  |  | 1.118 | 0.871-1.427 |  |  |  |  |  |  |
|  |  |  |  |  |  |  |  |  |  |  |  |  |  |  |  |  |  |
| **Mean number of adults living in the household** | 1.096 | 0.920-1.343 |  |  |  |  |  |  |  | 1.150 | 0.892-1.556 |  |  |  |  |  |  |
|  |  |  |  |  |  |  |  |  |  |  |  |  |  |  |  |  |  |
| **Mean number of children living in the household** | 1.060 | 0.823-1.294 |  |  |  |  |  |  |  | 1.098 | 0.778-1.469 |  |  |  |  |  |  |
|  |  |  |  |  |  |  |  |  |  |  |  |  |  |  |  |  |  |
| **Travel cost (N$)** |  |  |  |  |  |  |  |  |  |  |  |  |  |  |  |  |  |
| **Mean travel cost** | 1.080 | 0.242-2.205 |  | 0.973 | 0.266-2.747 |  | 0.978 | 0.121-1.877 |  | 1.382 | 0.190-4.039 |  | 1.090 | 0.231-4.27 |  | 1.621 | 0.071-6.112 |
| **75^th^ percentile travel cost** | 0.706 | 0.250-1.652 |  | 1.440 | 0.400-3.081 |  | **0.569** | **0.150-1.0** |  | 0.667 | 0.2-2.0 |  | 1.609 | 0.3-4.0 |  | **0.440** | **0.050-1.0** |
|  |  |  |  |  |  |  |  |  |  |  |  |  |  |  |  |  |  |
| **Travel time to a health care center** |  |  |  |  |  |  |  |  |  |  |  |  |  |  |  |  |  |
| **Mean travel time to a health care center** | **0.731** | **0.508-0.958** |  | 0.871 | 0.465-1.322 |  | **0.701** | **0.468-0.953** |  | **0.661** | **0.430-0.940** |  | 0.860 | 0.432-1.426 |  | **0.571** | **0.332-0.916** |
| **25^th^ percentile travel time to a health care center** | **0.823** | **0.5-1** |  | 1.006 | 0.125-2.0 |  | **0.786** | **0.5-1.0** |  | **0.763** | **0.333-1.0** |  | 0.998 | 0.125-2.0 |  | **0.651** | **0.286-1.0** |
| **75^th^ percentile travel time to a health care center** | 0.757 | 0.417-1.111 |  | 1.032 | 0.375-1.667 |  | **0.661** | **0.317-1.0** |  | 0.661 | 0.285-1.168 |  | 1.052 | 0.333-1.826 |  | **0.483** | **0.250-1.0** |
|  |  |  |  |  |  |  |  |  |  |  |  |  |  |  |  |  |  |
| **Proportion of individuals unable to access a health care center** | 0.927 | 0.754-1.085 |  |  |  |  |  |  |  | 0.903 | 0.683-1.126 |  |  |  |  |  |  |
|  |  |  |  |  |  |  |  |  |  |  |  |  |  |  |  |  |  |
| **Mean number of travel destinations** | **1.298** | **1.021-1.587** |  | 1.470 | 0.935-2.062 |  | 1.076 | 0.865-1.312 |  | **1.490** | **1.031-1.982** |  | 1.616 | 0.918-2.378 |  | 1.160 | 0.758-1.658 |
|  |  |  |  |  |  |  |  |  |  |  |  |  |  |  |  |  |  |
| **Proportion of individuals with at least one deceased child** | 0.944 | 0.541-1.366 |  |  |  |  |  |  |  | 0.949 | 0.449-1.605 |  |  |  |  |  |  |

Table N: Absolute difference between estimates based on mobile phone owners with reference to all participants or non-phone owners.

|  | **Reference: all participants** | | | | | | | |  | **Reference: non-phone owners** | | | | | | | |
| --- | --- | --- | --- | --- | --- | --- | --- | --- | --- | --- | --- | --- | --- | --- | --- | --- | --- |
|  | **Absolute**  **difference** | **95% CI** |  | **Women** | |  | **Men** | |  | **Absolute**  **difference** | **95% CI** |  | **Women** | |  | **Men** | |
|  |  |  |  | **Absolute**  **difference** | **95% CI** |  | **Absolute**  **difference** | **95% CI** |  |  |  |  | **Absolute**  **difference** | **95% CI** |  | **Absolute**  **difference** | **95% CI** |
| **Travel cost (N$)** |  |  |  |  |  |  |  |  |  |  |  |  |  |  |  |  |  |
| **Mean travel cost** | 29.639 | -117.213-283.567 |  | -2.782 | -164.504-364.706 |  | 28.393 | -143.923-283.012 |  | 42.497 | -163.217-387.515 |  | -2.561 | -199.558-431.067 |  | 53.677 | -240.737-516.183 |
| **75^th^ percentile travel cost** | -33.155 | -125.0-70.125 |  | 48.562 | -100.0-250.0 |  | -39.282 | -90.062-0 |  | -46.522 | -155.125-100.0 |  | 61.108 | -100.0-300.0 |  | **-98.130** | **-426.625-0** |
|  |  |  |  |  |  |  |  |  |  |  |  |  |  |  |  |  |  |
| **Travel time to a health care center** |  |  |  |  |  |  |  |  |  |  |  |  |  |  |  |  |  |
| **Mean travel time to a health care center** | **-1.585** | **-3.171--0.215** |  | -0.839 | -3.492-1.816 |  | **-1.667** | **-3.307--0.224** |  | **-2.253** | **-4.427--0.323** |  | -0.990 | -4.144-2.103 |  | **-3.074** | **-6.157--0.448** |
| **25^th^ percentile travel time to a health care center** | **-0.364** | **-1.0-0** |  | 0.001 | -2.0-2.5 |  | **-0.438** | **-1.0-0** |  | **-0.585** | **-2.0-0** |  | -0.056 | -2.0-2.5 |  | **-0.969** | **-2.5-0** |
| **75^th^ percentile travel time to a health care center** | -2.075 | -7.0-1.0 |  | -0.122 | -6.0-4.0 |  | **-2.807** | **-8.0-0** |  | -3.502 | -8.256-1.256 |  | -0.218 | -8.0-5.0 |  | **-5.431** | **-9.0-0** |
|  |  |  |  |  |  |  |  |  |  |  |  |  |  |  |  |  |  |
| **Mean number of travel destinations** | **0.707** | **0.048–1.372** |  | 0.838 | -0.116-1.741 |  | 0.229 | -0.428-0.908 |  | **0.996** | **0.069–1.831** |  | 0.984 | -0.151-2.009 |  | 0.394 | -0.850-1.524 |

Table O: Survey instrument

**1: Demographics**

- 1. Participant common name:

Participant nickname:

Participant big name: Surname (women=family name):

1.2 What year were you born? Approximate age:

1.3 Tribe: **Himba Tjimba Other __________**

1.4.2 Home village (where you spent *most* time in the past year): ____________________

1.4.3 If this is not your home village, why are you here? ___________________________

1.5 Marital status: **Not married married (mono) married (poly) divorced widowed**

1.6.0 Number of children: _______

1.7.1 Number of deceased children: ______

**2: Household resources**

2.1.1 How many people live in your house right now? **Adults_______ Children_________**

2.1.2 How many people live in your compound right now? **Adults_____ Children_________**

2.1.3 How many people sleep inside your house most nights? **Adults______Children______**

2.2.1 How many cattle do you have?

2.2.2 How many cattle does your compound have total, including your own?

2.3.1 How many goats & sheep do you have?

2.3.2 How many goats & sheep does your compound have total, including your own?

2.4.1 How many 50kg bags of maize did your garden produce last year?

2.4.2 When did the maize finish? **Month: Season:**

2.5.1 Will you inherit any cows?

2.5.2 Will you inherit any shoats?

2.6.1 For the past few months, what foods do you eat often?

2.6.2 For the past few months, what foods do you eat sometimes?

**3: Mobility**

3.1.0 Think of all the places you went to outside your village—places both very close and very far—in **the past year**. Please tell me about the five places you went to most frequently.

| Trip no. | Where did you go? (Village or town name. If more than one village with that name, specify village.) | Why did you go to this place? | How did you travel? | How long did it take you? | How many times? (If frequently, ask weekly or daily?) | How many nights did you stay? |
| --- | --- | --- | --- | --- | --- | --- |
| 3.1.1 |  |  |  |  |  |  |
| 3.1.2 |  |  |  |  |  |  |
| 3.1.3 |  |  |  |  |  |  |
| 3.1.4 |  |  |  |  |  |  |
| 3.1.5 |  |  |  |  |  |  |

**3.5.0 The movement of others:**

3.5.1 When do most of the visitors to this village tend to travel here?

**Rainy Dry Winter Anytime/doesn’t matter People never really visit here DK**

3.5.2 What are some of the reasons they come here? **DK**

**Because:**

3.6 When do most of the people who live here tend to travel away from this village?

**Rainy Dry Winter Anytime/doesn’t matter People never really leave here DK**

**4: Social network data**

**4.4** **Social capital questions**

4.4.2 List up to five people who would care for you or your family, or help you with practical work if you became too sick to do those things for yourself?

| **Common name, nickname, big name** | **Year/Age** | **Sex** | **Home village** | **Relationship** |
| --- | --- | --- | --- | --- |
|  |  |  |  |  |
|  |  |  |  |  |
|  |  |  |  |  |
|  |  |  |  |  |
|  |  |  |  |  |

4.4.3 List up to five people you could speak to about a private matter that you trust to give you good advice and not gossip about you.

| **Common name, nickname, big name** | **Year/Age** | **Sex** | **Home village** | **Relationship** |
| --- | --- | --- | --- | --- |
|  |  |  |  |  |
|  |  |  |  |  |
|  |  |  |  |  |
|  |  |  |  |  |
|  |  |  |  |  |

- 1. **Negative ties questions**

4.5.1 List up to five people whom you see frequently (about once a week or more) whom you feel expect things from you (resources, money, labor, sex) that makes you nervous.

| **Common name, nickname, big name** | **Year/Age** | **Sex** | **Home village** | **Relationship** | **What do they expect?** |
| --- | --- | --- | --- | --- | --- |
|  |  |  |  |  |  |
|  |  |  |  |  |  |
|  |  |  |  |  |  |
|  |  |  |  |  |  |
|  |  |  |  |  |  |

**8. Mobile-phone use questions**

8.1.1 Have you ever used a mobile phone? ____________

8.2.1 How many mobile phones do you own _________ **N**

8.2.2 What was the cost of each mobile phone you own? _________

8.2.3 How many months ago did you get your phone? ________

8.2.4 Do you share it: **No with family with friends with village**

8.3.1 How many SIM cards do you own, other than the one that is in your phone? ________

8.3.4 Do you share it: **No with family with friends with village**

8.4.1 Have you ever borrowed a mobile phone?

**NO Y borrowed from; relationship (all): _____________________________**

8.4.2 How often do you borrow a mobile phone?

**__x day __ x week __ x month __x season __ ever other _____**

8.5.1 Have you ever borrowed a SIM card without a phone?

**NO Y borrowed from; relationship (all): _____________________________**

8.5.2 How often do you borrow a SIM card?

**__ x day __ x week __ x month __x season __ ever other _____**

8.7 Name villages/cities where you or someone you know can go to get phone reception.

| Location (Village/City/Geographic Feature) |
| --- |
|  |
|  |
|  |
|  |
|  |

8.9.1 How many people in your household or compound own a mobile phone? ________

8.9.2 For the person you know the best, do they share it:

**No with family with friends with village**

**9-12. General Health History**

**9. Access to health care**

9.1.1 Where is the nearest place you can get clinic care? Village/town name:

9.1.2 How long does it take you to get there? Travel time in hours:

9.1.3 How would you get there? **Walk Car (whose?) Other:________________**

*9.2.0 Think about the most recent time you went to any clinic for your or another person’s health problem.*

9.2.1 When was the last time you went a clinic? **(season/year)**

9.2.2 Where was it? Village/town name:

9.2.3 Who required medical attention (who was the patient)?

9.2.3 Why did you go (symptoms)?

9.2.4 How much did the clinic cost?

9.2.4 How much travel to the clinic cost?

9.2.6 What did the nurse or doctor tell you was wrong? **Did not explain clearly** or:

**Herero: ______________________________ English:________________________**

9.2.6 What did they give you for your symptoms?

9.2.7 How do you feel about how the nurse/doctor treated you during your visit?

**Overall positive Neutral Overall negative**

9.3.1 Have you ever wanted to get clinic care but been unable to? **Y N DK**

9.3.2 If yes, when you’re sick, what are reasons that you don't go to the clinic?

**Expense distance time transportation no childcare too sick**

**Not sick enough dislike distrust**

**Other:___________________________________________**

**10. Measles and other rashes**

10.1.1 Have you ever had itchy red spots that look like this (show rash photos, circle if yes):

1. measles
2. chicken pox
3. rubella
4. scarlet fever
5. prickly heat

10.1.2 If YES, how old were you when you had:

1. measles
2. chicken pox
3. rubella
4. scarlet fever
5. prickly heat

10.2.1 Did you seek medical treatment for any: **YES NO DK**

If yes, which? 1 2 3 4 5

**Children’s measles**

10.4.1 How many of your children have had any of these rashes and

10.4.2 If YES, can you tell me:

| Rash | # children | Age at infection | season | Medical treatment:Y/N |
| --- | --- | --- | --- | --- |
| Measles |  |  |  |  |
| Chicken pox |  |  |  |  |
| Rubella |  |  |  |  |
| Scarlet Fever |  |  |  |  |
| Prickly Heat |  |  |  |  |

10.4.3 How many others had measles around the same time you or your child had measles?

**everyone many others few others no one else had measles then**

10.7.1 During which season do you see measles most? Circle all that apply

**Rainy Dry Winter None**

**11. Vaccines and Immunizations**

11.1.1 Have any vaccines ever been offered to you or others in the village?

**NO DK YES**

11.1.2 Do you know what were the vaccines were: **Can’t remember DK Yes**

11.1.3 If yes, I think it was: (anything you can remember, show photos of vaccinations)

| Year | Season | Delivery (shot, oral, etc) | From who? | Did you or child receive? |
| --- | --- | --- | --- | --- |
|  |  |  |  |  |
|  |  |  |  |  |
|  |  |  |  |  |
|  |  |  |  |  |

11.1.4 Have you ever been offered a vaccine and refused to get it? **__ YES NO**______

**12. Health Concerns: personal and community**

12.1.1 What are the diseases, health risks and dangers that are the biggest problem for you, your family, friends and your community *right now*?

12.2.1 What are the diseases, health risks and dangers that you most worry about for you, your family, friends and your community *for the future*?

**References**

1. Hazel A, Foxman B, Low BS. Herpes simplex virus type 2 among mobile pastoralists in northwestern Namibia. Ann Hum Biol. 2015;42(6):543–51.

2. White IR, Royston P, Wood AM. Multiple imputation using chained equations: Issues and guidance for practice. Stat Med. 2011;30(4):377–99.

3. Buuren S van, Groothuis-Oudshoorn K. mice: Multivariate Imputation by Chained Equations in R. J Stat Softw. 2011 Dec 12;45:1–67.

4. Marshall A, Altman DG, Holder RL, Royston P. Combining estimates of interest in prognostic modelling studies after multiple imputation: current practice and guidelines. BMC Med Res Methodol. 2009 Jul 28;9:57.

5. Rubin DB. Multiple imputation for nonresponse in surveys. Vol. 81. John Wiley & Sons; 2004.

6. Koelle DM, Wald A. Herpes simplex virus: the importance of asymptomatic shedding. J Antimicrob Chemother. 2000 Apr 1;45(suppl_4):1–8.

7. Brand J, Buuren S van, Cessie S le, Hout W van den. Combining multiple imputation and bootstrap in the analysis of cost-effectiveness trial data. Stat Med. 2019;38(2):210–20.
